# *CCR5* Gene Disruption via Lentiviral Vectors Expressing Cas9 and Single Guided RNA Renders Cells Resistant to HIV-1 Infection

**DOI:** 10.1371/journal.pone.0115987

**Published:** 2014-12-26

**Authors:** Weiming Wang, Chaobaihui Ye, Jingjing Liu, Di Zhang, Jason T. Kimata, Paul Zhou

**Affiliations:** 1 The Unit of Anti-Viral Immunity and Genetic Therapy, the Key Laboratory of Molecular Virology and Immunology, the Institut Pasteur of Shanghai, Chinese Academy of Sciences, Shanghai, China 200025; 2 Bio-X Institutes, Key Laboratory for the Genetics of Developmental and Neuropsychiatric Disorders (Ministry of Education), Shanghai Jiao Tong University, Shanghai, China 200030; 3 Department of Molecular Virology and Microbiology, Baylor College of Medicine, Houston, Texas 77030; Shanghai Medical College, Fudan University, China

## Abstract

CCR5, a coreceptor for HIV-1 entry, is a major target for drug and genetic intervention against HIV-1. Genetic intervention strategies have knocked down CCR5 expression levels by shRNA or disrupted the CCR5 gene using zinc finger nucleases (ZFN) or Transcription activator-like effector nuclease (TALEN). In the present study, we silenced *CCR5* via CRISPR associated protein 9 (Cas9) and single guided RNAs (sgRNAs). We constructed lentiviral vectors expressing Cas9 and CCR5 sgRNAs. We show that a single round transduction of lentiviral vectors expressing Cas9 and CCR5 sgRNAs into HIV-1 susceptible human CD4^+^ cells yields high frequencies of *CCR5* gene disruption. *CCR5* gene-disrupted cells are not only resistant to R5-tropic HIV-1, including transmitted/founder (T/F) HIV-1 isolates, but also have selective advantage over *CCR5* gene-undisrupted cells during R5-tropic HIV-1 infection. Importantly, using T7 endonuclease I assay we did not detect genome mutations at potential off-target sites that are highly homologous to these CCR5 sgRNAs in stably transduced cells even at 84 days post transduction. Thus we conclude that silencing of *CCR5* via Cas9 and CCR5-specific sgRNAs could be a viable alternative strategy for engineering resistance against HIV-1.

## Introduction

Entry of HIV-1 into human CD4 T cells is initiated with the binding of the viral envelope protein gp120 to the CD4 receptor on the cell surface. Subsequently, a conformational change in gp120 allows its interaction with a coreceptor, CCR5 or CXCR4. Coreceptor binding activates gp41, enabling it to mediate fusion of the viral and cellular membranes and the release of the viral core into the cytoplasm. Depending on coreceptor usage, HIV-1 variants are classified as being CCR5 (R5), CXCR4 (X4), or dual-tropic [1]. For reasons that are still not completely understood, HIV-1 founder viruses transmitted across mucosal surface by sexual contact, by maternal-infant exposure, and by percutaneous inoculation are all R5 viruses [2]. Furthermore, individuals with a homozygous CCR5Δ32 deletion are highly resistant to HIV-1 infection [3]–[5]. As a result, CCR5 has been one of major targets for drug and genetic intervention against HIV-1 infection [6].

Initially, genetic intervention focused on phenotypic knock-down of CCR5 expression levels using intracellular antibodies [7], transdominant mutants [8], ribozymes [9] and siRNAs [9], [10]. More recently, disruption of CCR5 at the genomic level has been studied using zinc finger nucleases (ZFNs) [11]–[14] and TALE nuclease (TALEN) [15]. *CCR5* disruption was obtained following a single round of transduction with the adenovirus vectors expressing CCR5-ZFN or electroporation of a plasmid DNA expressing CCR5-ZFN [11], [13]. When CCR5-ZFN-transduced cells were infected *in vitro* with R5-tropic HIV-1 isolates, a two-fold enrichment of the *CCR5*-modified cells was observed at the end of the culture period [11]. Moreover, a significant increase of *CCR5*-modified cells and lower plasma viremia were observed in HIV-1 infected NOG mice engrafted with CCR5-ZFN-transduced T cells but not NOG mice engrafted with mock-transduced T cells [11]. Currently CCR5-ZFN-modified, *ex vivo* expanded autologous T cells are in Phase I clinical trials [10], [16].

Bacterial and archaeal CRISPR (clustered regularly interspaced short palindromic repeats) systems rely on CRISPR RNAs (crRNAs) in complex with CRISPR-associated (Cas) proteins to direct degradation of complementary sequences present within invading viral and plasmid DNA [17], [18]. In *in vitro* reconstitution of the *Streptococcus pyogenes* type II CRISPR system, single guide RNAs (sgRNA, i.e. crRNA-tracrRNA fusion chimeras) are sufficient to direct the Cas9 endonuclease to specifically cleave target DNA sequences matching the crRNA [19]. This two-component system enables efficient genome editing in eukaryotic cells [20]-[23] and even in model organisms [20], [24]–[31].

Although the two-component sgRNA/Cas9 system has many advantages, such as ease of design and construction, low cost, possibility for highly multicomplexed modifications and efficient site-specific targeting, whether this system could become a viable alternative to ZFN and TALEN in genotypic disruption of *CCR5* depends on its efficiency and target sequence specificity. Recently, Cho *et al.* showed high frequencies of indels within *CCR5* of the K562 cell line co-transfected with DNA plasmids encoding Cas9 and 2 of 28 CCR5 sgRNAs, but no indels at any of potential off-target sites to these 2 CCR5 sgRNAs [32]. However, when additional 9 CCR5 sgRNAs were tested, off-target mutations at *CCR2* sequences that bear one nucleotide mismatch to 6 CCR5 sgRNAs were detected [33]. Cradick *et al.* showed that although high frequencies of indels occurred within *CCR5* in 293 cells co-transfected with DNA plasmids encoding Cas9 and 5 different CCR5 sgRNAs, off-target indels at *CCR2* gene were detected in cells transduced with just 2 of 5 CCR5 sgRNAs [34]. More recently, Ye *et al*. combined TALENs or sgRNA-Cas9 with piggyBac technology to generate CCR5Δ32 deletion mutant in induced pluripotent stem cells (iPSCs). They then differentiated the modified iPSCs into monocytes/macrophages and demonstrated that these cells were resistant to HIV-1 challenge [35]. Although these studies showed that *CCR5* gene disruption can be generated in 293 and K562 cells and iPSCs and modified iPSCs, when differentiated into monocytes/macrophages, were resistant to HIV-1 challenge, the efficiency and the specificity of individual sgRNAs that target different CCR5 sequence segments in human CD4 T cells, the major cell targets for HIV-1, remain to be carefully evaluated.

In the present study, we examined *CCR5* gene disruption using lentiviral vectors expressing Cas9 and CCR5 sgRNAs. Here we report that a single round co-transduction of these lentiviral vectors into HIV-1 susceptible TZM.bl and CEMss-CCR5 cells results in high frequencies of human *CCR5* gene disruption. *CCR5*-modified cells not only are resistant to infection by R5-tropic HIV-1 including transmitted founder (T/F) viruses, but also have a selective advantage over cells carrying wild type *CCR5* alleles during R5-tropic HIV-1 infection. Importantly, using T7 endonuclease I assay we did not detect indels at 12 potential off-target sites that are highly homologous to these CCR5 sgRNAs even at 84 days post transduction. Finally, we showed that a single round transduction of a single lentiviral vector expressing both CCR5 sgRNA and Cas9 also efficiently disrupts *CCR5* gene in CEMss-CCR5 cells. Thus, we conclude that *CCR5* gene disruption using lentiviral vectors expressing Cas9 and specific CCR5 sgRNAs may be a viable alternative genetic intervention strategy against HIV-1.

## Materials and Methods

### Cell lines and viruses including transmitted/founder (T/F) HIV-1 isolates

The packaging cell line 293T was purchased from Invitrogen Life Technologies and maintained in complete DMEM medium [i.e. high glucose DMEM supplemented with 10% FBS, 2 mM L-glutamine, 1 mM sodium pyruvate, penicillin (100 U/ml), streptomycin (100 µg/ml)] plus G418 (500 µg/ml) (Invitrogen Life Technologies). TZM.bl cells [36] were obtained from the NIH AIDS Research and Reference Reagent Program (ARRRP, Germantown, MD) and maintained in complete DMEM. CEMss-CCR5 cells were generated previously [37] and maintained in complete DMEM medium.

Infectious molecular clones of HIV-1 strain Bru3 and Bru-Yu2 and infectious molecular clones of transmitted/founder (T/F) HIV-1 isolates, pWITO, pCH040, pTHRO, pREJO, and pCH106 [38] were obtained from ARRRP, Germantown, MD. To generate infectious Bru3, Bru-Yu2 and (T/F) viruses, 293T cells were transfected with the infectious molecular clones as described before [37]. Culture supernatants containing Bru3, Bru-Yu2 and infectious (T/F) viruses were harvested and titrated onto TZM.bl cells as described [39].

### pRRL-Cas9-HA-NLS, pRRL-U6-sgRNA and pRRL-CR2 sgRNA-Cas9/EGFP lentiviral transfer constructs

Human codon optimized fusion gene encoding *S. Pyogenes* Cas9, HA tag and SV40 NLS (nuclear localization signal) was synthesized by a commercial service company (Genescript, Nanjing, China) and verified by sequencing. The correct sequence was inserted into Bam H1 and Sal I sites of the third generation of lentiviral transfer vector pRRL [40]. The resulting vector was designated as pRRL-Cas9-HA-NLS. Human codon optimized fusion genes containing the U6 promoter and sequence transcribing sgRNAs (CR1, CR2, CR3 or GF1) were also synthesized by the same company and verified by sequencing. The correct sequences were inserted into Xho I of the same transfer vector. The resulting vectors were designated as pRRL-sgRNAs (CR1, CR2, CR3 or GF1).

To construct a single lentiviral transfer vector expressing both CR2 sgRNA and Cas9, the fusion gene Cas9/HA/NLS/2A/EGFP driven by EF1α promoter was synthesized and inserted into BamHI and SalI sites of pRRL-sgRNA CR2. The resulting vector was designated as pRRL-CR2 sgRNA-Cas9/EGFP.

### Recombinant lentivirus production

Recombinant lentiviruses were generated as described before [39]. Briefly, 4×10^6^ 293T cells were seeded onto P-100 dish in 10 ml complete DMEM. After overnight culture, cells were co-transfected with 14 *u*g lentiviral transfer vector [one of pRRL-Cas9-HA-NLS,pRRL-U6-sgRNAs (CR1, CR2, CR3 or GF1) or pRRL-CR2 sgRNA-Cas9/EGFP], 7.5 ug of packaging construct delta 8.9 and 3 ug of plasmid encoding the VSV-G envelope (pLP/VSVG) using a calcium phosphate precipitation method as described [41]. Sixteen hours later, culture supernatants were removed and replaced with fresh complete DMEM plus 1 mM sodium butyrate (Sigma). Eight hours later, supernatants were again removed and replaced with fresh DMEM plus 10% FBS. After another 20 hours, the culture supernatants were harvested, centrifuged at 2,000 rpm for 10 minutes and filtered through 0.45 µm filter. The filtered supernatants were concentrated by ultra-centrifugation as described before [39]. The lentiviral vector pellets were resuspended in a small volume of RPMI 1640 and stored in aliquots at −80°C freezer. Lentiviral vector titers were determined as we previously described [39].

### Generation of stably transduced TZM.bl and CEMss-CCR5 cell lines co-expressing Cas9-HA-NLS and sgRNAs

To transduce TZM.bl cells, 5×10^4^ TMZ.bl cells per well were seeded onto 24 well plate. After overnight culture, cells were co-transduced with 2×10^6^ TU of lentiviral vectors expressing Cas9-HA-NLS and sgRNA CR1, CR2 or CR3 (therefore, MOI of 40) in the presence of 8 µg/ml of polybrene. As a control, TZM.bl cells were co-transduced with 2×10^6^ TU of lentiviral vectors expressing Cas9-HA-NLS and sgRNA GF1 in the presence of 8 µg/ml of polybrene. Seven days after the transduction CCR5 expression on the surface of transduced TZM.bl cells was measured by antibody staining followed by FACS analysis (see below). CCR5 negative cell populations were sorted out by PE-conjugated anti-CCR5 antibody staining followed by FACS sorting (see below).

To transduce CEMss-CCR5 cells, 1×10^5^ cells per well were seeded onto 24 well plate. After overnight culture, cells were co-transduced with 2×10^6^ TU of lentiviral vectors expressing Cas9-HA-NLS and sgRNA CR2 or GF1 (therefore, MOI of 20) or transduced with 2×10^6^ TU of a single lentiviral vector expressing both CR2 sgRNA and Cas9 (therefore, MOI 20) in the presence of 8 µg/ml of polybrene. Seven days after the transduction CCR5 expression on the surface of transduced CEMss-CCR5 cells was measured by antibody staining followed by FACS analysis (see below). CCR5 negative cell populations were sorted out by PE-conjugated anti-CCR5 antibody staining followed by FACS (see below).

### FACS analysis

To analyze cell surface expression of CCR5, CXCR4 and CD4, 2×10^5^ mock- and Cas9-HA-NLS and sgRNAs (CR1, CR2, CR3 or GF1)-transduced TZM.bl cells were incubated with a PE-conjugated mouse anti-human CCR5 (BD Biosciences), PE-conjugated mouse anti-CXCR4 (Becton Dickinson) or APC-conjugated mouse anti-CD4 (Miltenyi) antibody for 45 min on ice. Cells then were washed twice with FACS buffer (PBS containing 1% BSA and 0.02% NaN_3_) and then washed twice with FACS buffer and fixed with 1% formaldehyde in 0.5 ml of FACS buffer. FACS analysis was performed on LSRII (Becton Dickinson, Mountain View, CA).

To isolate CCR5 negative cell populations from TZM.bl cells co-transduced with lentiviral vectors expressing Cas9-HA-NLS and sgRNAs CR1, CR2 or CR3 or from CEMss-CCR5 cells co-transduced with lentiviral vectors expressing Cas9-HA-NLS and sgRNAs CR2, transduced cells were incubated with a mouse PE-conjugated anti-human CCR5 antibody (BD Biosciences) for 45 min on ice and then washed twice with PBS containing 1% BSA. CCR5 negative cell populations were sorted using an Arial II cell sorter (Becton Dickinson).

### Genome analysis–T7 endonuclease I assay and sequencing

Efficiency of CCR5 genome disruption was measured by performing PCR amplification with a set of prime pairs across crRNA target sequences followed by the digestion with T7 endonuclease I (NEB). The latter detects heteroduplex formation of double stranded DNA. Briefly, genomic DNA were extracted from CCR5^-^/CR1, CCR5^-^/CR2 and CCR5^-^/CR3 and mock-transduced TZM.bl or CEMss-CCR5 cells and subject to PCR amplification with a set of prime pairs across crRNA target sequences (see S1 Table). The resulting PCR products were digested with T7 endonuclease I and resolved by agarose gel electrophoresis.

To analyze potential off-target mutations, we built the human exon region reference as described by Mali *et al*. [21]. The query sequences were mapped to the human exon region reference database with max mismatch settings at 0 (no mismatch) and 1 (a single mismatch) within the 3′ end 13 nucleotide seed and PAM (NGG) sequences of CR1, CR2 and CR3, respectively. A total 12 potential off-target sequences were identified: 3 single mismatches (AKAP9, ULK1 and MED16) to CR2 and 9 single mismatches (SH2D5, ASB9P1, PRRT1, LINC00265, ENDOV, NR2F1, ASB9, CLPP and SOBP) to CR3. Genomic DNA were then extracted from CCR5^-^/CR2 and CCR5^-^/CR3 at 21 and 84 days post transduction along with mock-transduced TZM.bl cells and subject to PCR amplification with 12 prime pairs listed in S2 Table followed by T7 endonuclease I assay as described above.

To further analyze *CCR5* gene disruption, the above PCR products were cloned into a TA cloning vector and sequenced. The indels of the *CCR5* gene were identified by comparing with the wild type CCR5 sequence.

### Generation of HIV-1 pseudotypes and a single-cycle infectivity assay

To generate pseudotypes with the HIV-1 vector, 4×10^6^ 293T packaging cells were co-transfected with 10 µg of HIV-1-luciferase transfer vector [42] and 10 µg of DNA plasmid encoding one of HIV-1 envelopes pDOL, CNE3, CNE11, CNE30 and Yu-2 or control retroviral envelope 10A1 using a calcium phosphate precipitation method [43]. DNA plasmids encoding HIV-1 envelopes pDOL and Yu-2 were obtained from ARRRP. DNA plasmids encoding CNE3, CNE11 and CNE30 were obtained from Dr. Linqi Zhang at the Tsinghua University. HIV-1 envelope HIV-1 envelopeYu-2 is derived from a R5 tropic clade B virus [44]. HIV-1 envelope pDOL is derived from an X4 tropic clade B virus [45]. HIV-1 envelope CNE11 is derived from a R5 tropic CRF01_AE recombinant. HIV-1 envelope CNE3 is derived from a R5 tropic clade B′ virus. HIV-1 envelope CNE30 is derived from a R5 tropic clade CRF07_B′C recombinant [46]. The pseudotype-containing supernatants were harvested and stored in aliquots at −80°C degree freezer. The amount of HIV-1 p24 in collected supernatants was measured by ELISA.

In a single-cycle assay to measure the infectivity of HIV-1 pseudotypes or wild type viruses including transmitted founder (T/F) viruses, 1×10^4^ mock-, Cas9-HA-NLS and sgRNAs (CR1, CR2 or CR3)-transduced TZM.bl cells were transduced with HIV-1 pseudotype-containing supernatants equivalent to relative luciferase activity 200,000 to 500,000 or wild type viruses including transmitted founder (T/F) viruses at the MOI of 0.2 overnight. Cells were then washed twice with PBS and cultured in complete DMEM medium for 2 days. Cells were then washed once with PBS and lysed in 100 µl of lysis buffer. Luciferase activity in 50 µl of cell suspensions was measured by a BrightGlo Luciferase assay according to the manufacturer's instruction (Promega).

### Cell growth assay

To compare cell growth, CCR5^-^/CR1, CCR5^-^/CR2 and CCR5^-^/CR3 and mock-transduced TZM.bl or CEMss-CCR5 cells (1×10^5^ per sample) were labeled with 20 µM CellTrace Far Red DDAO-SE (DDAO, Life Technology) for 40 minutes and then washed extensively with DMEM to remove free DDAO. At 0, 12, 24 and 36 hours post culture a portion of cells was harvested and fixed with 4% formaldehyde. Fluorescent intensity of DDAO was measured by FACS analysis using LSR II.

### HIV-1 challenge experiments

To test whether CCR5 genome-disrupted cells were resistant to R5-tropic HIV-1 infection, sorted CCR5^-^/CR2 and CCR5^+^/GF1 CEMss cells were infected with either X4-tropic HIV-1 Bru-3 or R5-tropic HIV-1 Bru-Yu2 at the MOI of 0.2 at 37°C, 5% CO_2_ for 2 hours. Cells were then washed twice with complete DMEM and resuspended in fresh complete DMEM. After the challenge, cells and culture supernatants were collected every 3 days and replenished with fresh medium for a total of 21 days. The amount of HIV-1 gag p24 in culture supernatants was measured by ELISA as instructed by manufacturer (Zepto Metrix).

To test whether potential selective advantage of CCR5 genome-disrupted over CCR5 genome-undisrupted cells during R5-tropic HIV-1 infection, we mixed 1×10^5^ CCR5^-^/CR2 and 9×10^5^ mock-transduced CEMss-CCR5 cells and then challenged cell mixture with either X4-tropic HIV-1 Bru-3 or R5-tropic HIV-1 Bru-Yu2 at the MOI of 0.2 at 37°C, CO_2_ for 4 hours. Cells were then washed twice with complete DMEM. After the challenge, cells and culture supernatants were collected every 3 days and replenished with fresh medium for a total of 18 days. At day 6 and 18 a portion of cells were stained with anti-CCR5 and anti-CD4 antibodies followed by FACS analysis (see above) and genomic DNA were extracted from another portion of cells and subjected to T7 endonuclease I assay (see above).

## Results

### Lentiviral vectors expressing the Cas9 and sgRNAs efficiently disrupt CCR5

**Figure 1:**
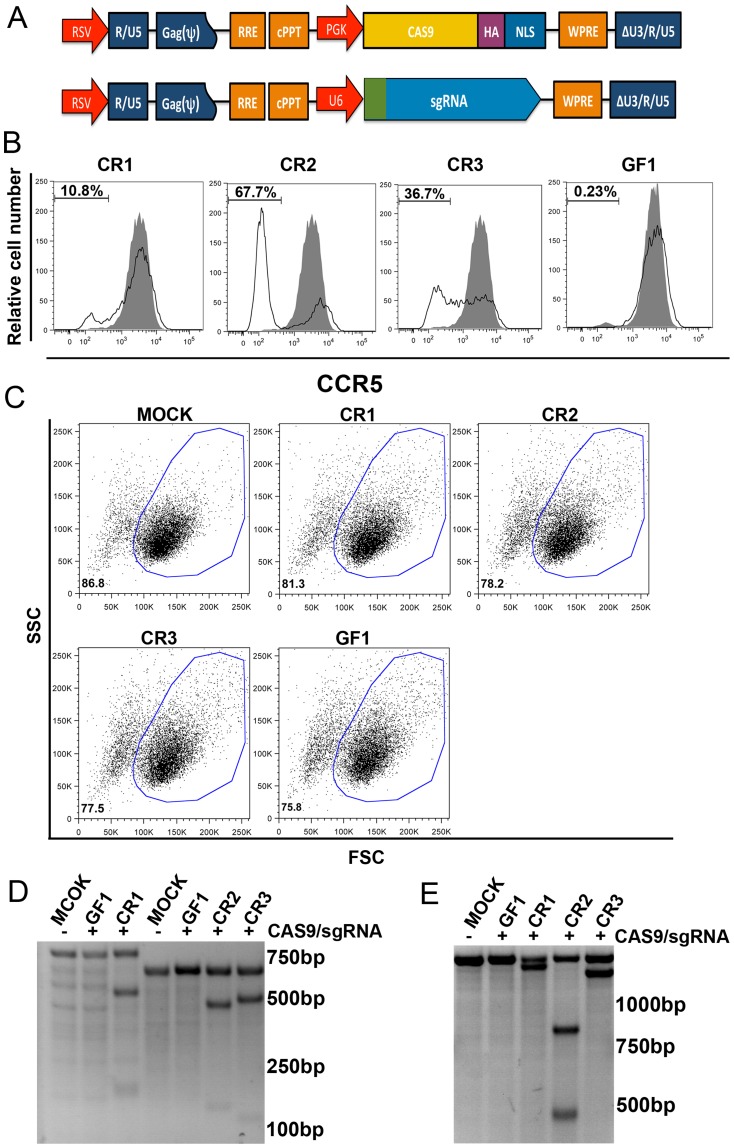
*CCR5* gene disruption by RNA-guided Cas9 endonuclease in transduced TZM.bl cells. A. Schematic diagrams of lentiviral transfer vectors containing Cas9 endonuclease or sgRNAs CR1, CR2, Cr3 and GF1. HA: HA epitope tag; NLS: nuclear localization signal; PGK: promoter sequence derived from phosphoglycerate kinase-1; U6: promoter sequence derived from polymerase III U6. B. Cell surface expression of CCR5 on TZM.bl cells co-transduced with lentiviral vectors expressing Cas9-HA-NLS and sgRNAs CR1, CR2, CR3 or GF1. Transduced (open curves) and mock-transduced (shaded curves) cells were stained with anti-CCR5 antibody followed by FACS analysis. C. FSC and SSC analysis mock-transduced TZM.bl cells and TZM.bl cells transduced with lentiviral vectors expressing Cas9-HA-NLS fusion protein and sgRNAs CR1, CR2, CR3 or GF1, respectively. Percentages of gated (live) cells are shown at the lower and left corner of the figures. D. *CCR5* gene disruption analysis by T7EI assay using prime pairs CR1/F990-CR1/R1750 and CR2/3F593-CR2/3R1254 (S1 Table). E. *CCR5* gene disruption analysis by T7EI assay using prime pair CR1/2/3F2559-CR1/2/3R3893 (S1 Table).

To disrupt *CCR5* in HIV-1 susceptible CD4^+^ cells we constructed a third generation lentiviral vector to express: 1) a Cas9-HA-NLS fusion protein driven by an internal PGK promoter; or 2) one of three sgRNAs to CCR5 (designated as CR1, CR2 and CR3), or a sgRNA to GFP (designated as GF1) driven by a RNA polymerase III U6 promoter (Fig. 1A and S1 Fig.). The HA-tag was to facilitate detection and the NLS (nuclear localization signal) was to target the Cas9 protein to the nucleus. The lentiviral vector expressing Cas9-HA-NLS fusion protein was co-transduced into the HIV-1-susceptible cell line TZM.bl with the vectors expressing sgRNA CR1, CR2, CR3 or GF1 as described [37]. Seven days after the transduction, the levels of CCR5 expression on the surface of the transduced TZM.bl cells were determined by staining with fluorescent anti-CCR5 (Becton Dickinson) or isotype-control antibody (eBioscience) followed by FACS analysis. Fig. 1B shows that 10.8%, 67.7% and 36.7% of TZM.bl cells co-transduced with lentiviral vectors expressing Cas9-HA-NLS fusion protein and sgRNAs CR1, CR2 or CR3, respectively, were negative for CCR5 surface expression. In contrast, no reduction of CCR5 expression was observed in cells co-transduced with vectors expressing the Cas9-HA-NLS and GF1 control. FSC and SSC analysis shows that 75.8 to 81.3% of TZM.bl cells transduced with lentiviral vectors expressing Cas9-HA-NLS fusion protein and sgRNAs CR1, CR2, CR3 or GF1 were live cells, which was slightly lower than mock-transduced cells (Fig. 1C).

Since TZM.bl cells are originally derived from HeLa cells co-transduced with retroviral vectors expressing human *CD4* and *CCR5* transgenes as well as HIV-1 LTR-driven reporter genes [36], the genome of TZM.bl cells contains both endogenous *CCR5* alleles and an integrated *CCR5* transgene sequence. Besides the normal diploid chromosome 3, which contains *CCR5*, the genome of HeLa cells also contains a t(1∶3) and t(3∶5) interchromosomal translocation [47]. Therefore, in order to examine RNA-guided *CCR5* gene modification, we designed three *CCR5* primer pairs. Primer pair CR1F990 and CR1R1750 (S1 Table) amplifies sequences in exon 3 of *CCR5* containing the CR1 target sequence. This is present in both the endogenous *CCR5* gene and the integrated *CCR5* transgene. Primer pair CR2/3F593 and CR2/3R1254 (S1 Table) amplifies sequences in exon 3 of *CCR5* that include the CR2 and CR3 target sequences. The sequences are also present in both the endogenous *CCR5* gene and the integrated *CCR5* transgene. Primer pair CR1/2/3F2559 and CR1/2/3R3893 (S1 Table) amplifies sequences in intron 2/exon 3 of only the endogenous *CCR5* gene. It contains the CR1, CR2 and CR3 sequences. After PCR amplification, T7 endonuclease I assay was performed. Fig. 1 D and E show that using each primer pair set, *CCR5* gene disruption was observed in cells co-transduced with lentiviral vectors expressing Cas9-HA-NLS and CR1, CR2 or CR3, but not GF1 and mock-transduced TZM.bl cells.

### Heterogeneity of CCR5 gene disruption via lentiviral vectors expressing the Cas9 and sgRNAs

**Figure 2:**
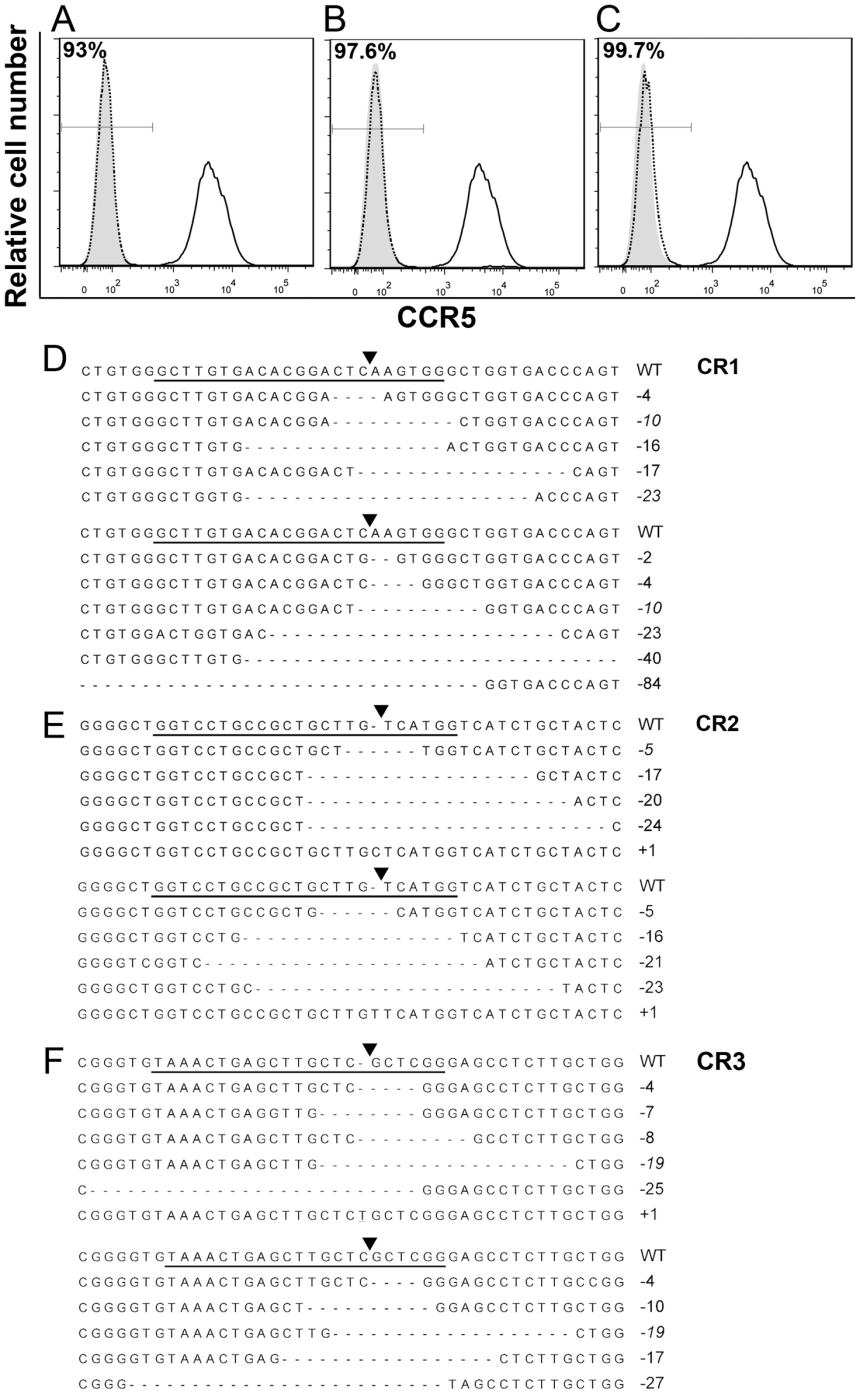
*CCR5* gene disruption by RNA-guided Cas9 endonuclease in sorted CCR5-/CR1, CCR5-/CR2 and CCR5-/CR3 cells by Sanger sequencing analysis. A-C. Cell surface expression of CCR5 on sorted CCR5-/CR1 (A), CCR5-/CR2 (B) and CCR5-/CR3 (C) TZM.bl cells as compared to mock-transduced TZM.bl cells. D-F. Representative Sanger sequencing of CCR5 target sites by CR1 (D), CR2 (E) or CR3 (F) in co-transduced TZM.bl cells. The full list of Sanger sequencing of CCR5 target sites by CR1, CR2 or CR3 is shown in S2 Fig. For each *CCR5* site targeted by CR1 (D), CR2 (E) or CR3 (F), the upper panel shows representative Sanger sequencing of targeted CCR5 sequences amplified by a prime pair that covers both the endogeous *CCR5* gene and transgene. The lower panel shows representative Sanger sequencing of targeted *CCR5* sequences by a prime pair that only covers the endogenous *CCR5* gene.

We next sorted CCR5 negative cell populations in cells co-transduced with lentiviral vectors expressing Cas9-HA-NLS and CR1, CR2 or CR3 by FACS. After sorting by FACS, CCR5 expression, *CCR5* gene disruption, off-target mutations and resistance to HIV-1 infection were investigated. Fig. 2A-C show that no CCR5 expression was detected on the surface of sorted *CCR5* ^-^ cell populations (referring to as CCR5^-^/CR1, CCR5^-^/CR2 and CCR5^-^/CR3 cells).

To analyze *CCR5* gene sequences, we isolated genomic DNA from CCR5^-^/CR1, CCR5^-^/CR2 and CCR5^-^/CR3 cells amplified the target sequences by PCR, and sequenced the PCR products. We found that substantial indels were detected in *CCR5* sequences isolated from CCR5^-^/CR1, CCR5^-^/CR2and CCR5^-^/CR3 cells (Fig. 2D to F and S2 Fig.). In CCR5^-^/CR1 cells, a total of 19 different deletion mutants were identified using the prime pair CR1F990 and CR1R1750 (S1 Table). The deletions ranged from -2 to -108 nucleotides. Using the prime pair CR1/2/3F2559 and CR1/2/3R3893, a total of 15 deletion mutants were detected. The deletions ranged from -2 to -839 nucleotides. All these mutants have the deletion matching to CR1 (Fig. 2D and S2 Fig.). In CCR5^-^/CR2 cells, a total of 9 different indels mutants were identified using the prime pair CR2F651 and CR2R820 (S1 Table). Most of the mutants contained deletions of −5 to −24 nucleotides. One mutant had an insertion of +1 nucleotide. Using the prime pair CR1/2/3F2559 and CR1/2/3R3893, a total of 16 indels mutants were detected. Most mutants contained deletions. These ranged in size from −5 to −426 nucleotides. One mutant had a +1 nucleotide insertion. Two deletion/insertion mutants were identified. These had -1 nucleotide/+1 nucleotide mutations and −2 nucleotides/+2 nucleotide mutations, respectively. All these mutants had the deletion/insertion matching to CR2 (Fig. 2E and S2 Fig.). Finally, using the prime pair CR3F1061 and CR3R1203 (S1 Table), a total of 13 deletion mutants with deletions of −2 to −25 nucleotides and 6 insertion mutants (all with +1 nucleotide) were identified in CCR5^-^/CR3 cells. Using the prime pair CR1/2/3F2559 and CR1/2/3R3893, a total of 20 indels mutant were detected. The deletion mutants contained deletions of −4 to -199 nucleotides. Among 8 insertion mutants, six mutants had +1 nucleotide and two had +2 nucleotides. One deletion/insertion mutant had −1 nucleotide, +2 nucleotides and another −7 nucleotides. All these mutants had the deletion/insertion matching to CR3 (Fig. 2F and S2 Fig.). Thus, these results clearly demonstrate a high level of human *CCR5* gene disruption in the endogenous *CCR5* gene and the integrated *CCR5* transgene via lentiviral vectors expressing Cas9 and CR1, CR2 or CR3.

### CCR5 genome-disrupted cells are resistant to R5-tropic HIV-1 infection

**Figure 3:**
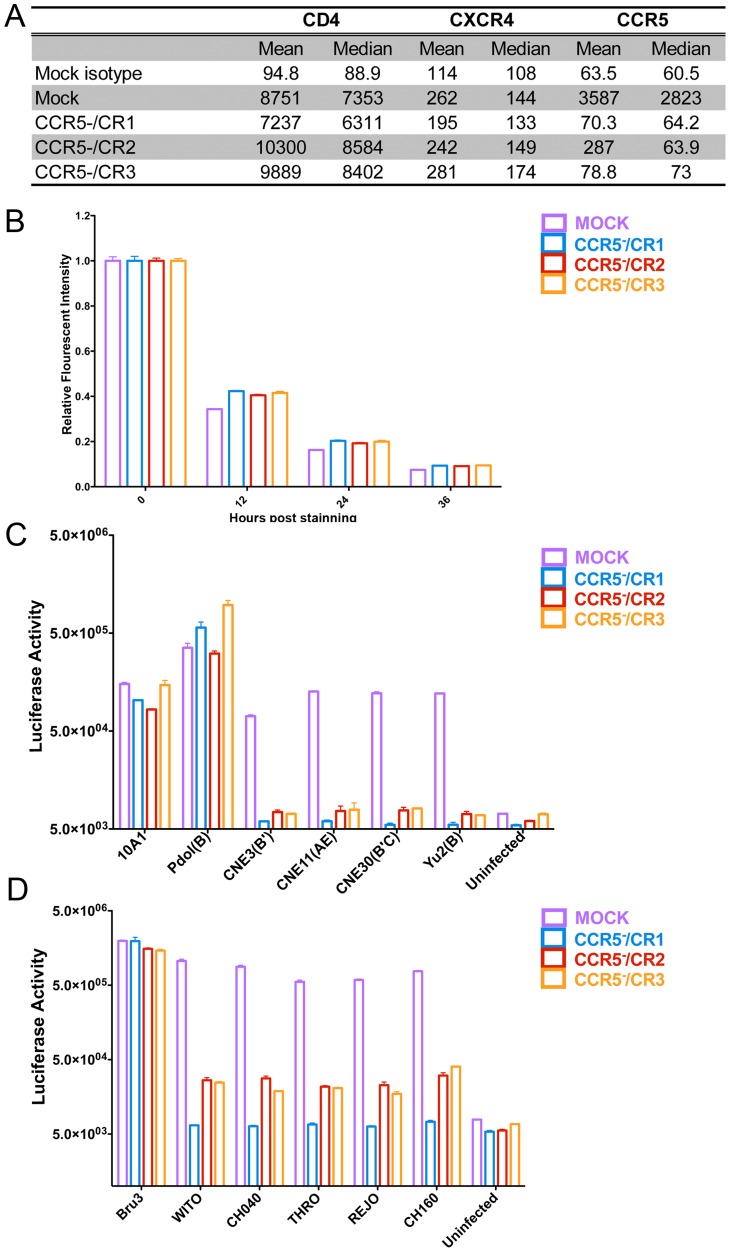
*CCR5* gene-disrupted cells are resistant to R5-tropic HIV-1 infection. A. Mean and median values of fluorescence intensity of cell surface expression of CD4, CXCR4 and CCR5 in CCR5-/CR1, CCR5-/CR2 and CCR5-/CR3 cells as compared to mock-transduced TZM.bl cells. B. Relative fluorescent intensity of DDAO-labeled CCR5-/CR1, CCR5-/CR2 and CCR5-/CR3 cells as compared to mock-transduced TZM.bl cells at 0, 12, 24 and 36 hours post culture. C. RLA of CCR5-/CR1, CCR5-/CR2 and CCR5-/CR3 cells as compared to mock-transduced TZM.bl cells infected with HIV-1 pseudotypes and the 10A1 MLV pseudotype control. D. RLA of CCR5-/CR1, CCR5-/CR2 and CCR5-/CR3 cells as compared to mock-transduced TZM.bl cells infected with HIV-1 Bru3 or transmitted founder (T/F) viruses.

Because CCR5, CXCR4 and CD4 expression or differences in cell growth could affect HIV-1 infection, we compared CXCR4 and CD4 expression and cell growth of CCR5^-^/CR1, CCR5^-^/CR2 and CCR5^-^/CR3 cells with those of mock-transduced TZM.bl cells. No significant difference in CD4 and CXCR4 expression (Fig. 3A) and in cell growth (Fig. 3B) was found among these cells.

To investigate their susceptibility or resistance to HIV-1 infection, CCR5^-^/CR1, CCR5^-^/CR2 and CCR5^-^/CR3 cells along with mock-transduced TZM.bl cells were infected with a panel of 5 HIV-1 pseudotypes and one retroviral control in a single-round infectivity assay [37], [41]. The 5 HIV-1 pseudotypes express the envelope proteins (Env) of subtypes B, B′, B/C and A/E [42], [46], [48]–[50]. Among them, Env pDOL is X4-tropic and the CNE3, CNE11, CNE30 and Yu2 Env proteins are R5-tropic. Control 10A1 envelope was derived from the 10A1 murine leukemia virus [51]. Fig. 3C shows that although all these cells exhibit similar relative luciferase activity (RLA) when infected with 10A1 and HIV-1 X4-tropic pDOL pseudotype viruses, significantly lower RLA was observed in the CCR5^-^/CR1, CCR5^-^/CR2 and CCR5^-^/CR3 mutant cells than in mock-transduced TZM.bl cells after infection with the four different R5-tropic Env pseudotyped viruses.

To further test the effect of the *CCR5* gene disruption on HIV-1 infection, we compared resistance of the CCR5^-^/CR1, CCR5^-^/CR2 and CCR5^-^/CR3 with that of mock-transduced TZM.bl cells to transmitted founder (T/F) HIV-1 isolates. Fig. 3D shows that although all these cells exhibit similar RLA when infected with HIV-1 X4-tropic Bru3, when infected with the five R5-tropic transmitted founder (T/F) HIV-1 isolates, WITO, CH040, THRO, REJO and CH106 [38], significantly lower RLA was found in CCR5^-^/CR1, CCR5^-^/CR2 and CCR5^-^/CR3 cells than in mock-transduced TZM.bl cells. Taken together, we conclude that cells with disrupted *CCR5* genes are highly resistant to R5-tropic HIV-1 infection.

### No mutation was detected at potential off-target sites that are highly homologous to CR2 and CR3

**Figure 4:**
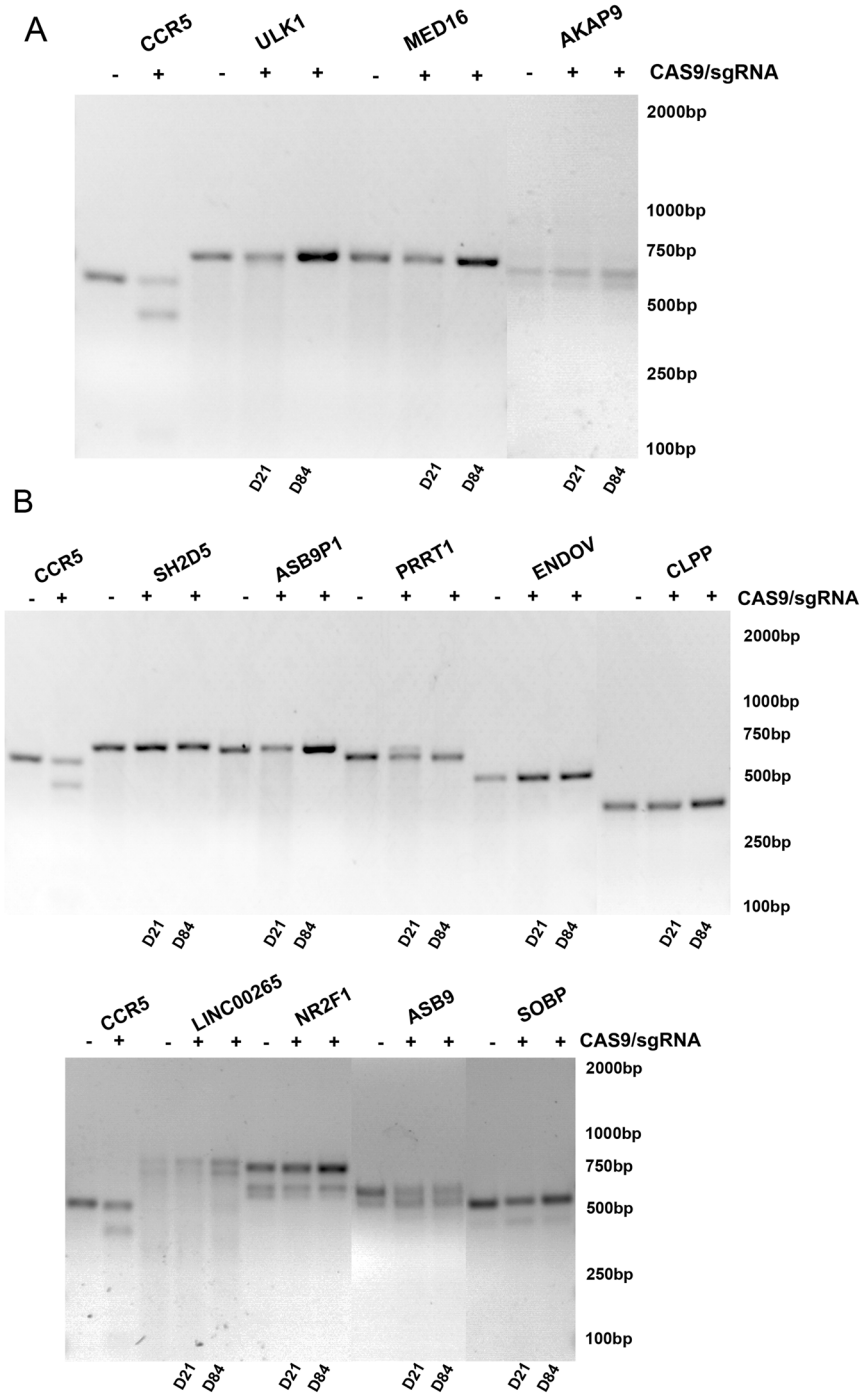
T7 endonuclease I (T7EI) analysis of potential off-target loci. A. T7EI analysis of three potential off-target loci (AKAP9, ULK1 and MED16) to CR2 as well as on-target locus CCR5. “–”: genomic DNA isolated from mock-transduced TZM.bl; “+”: genomic DNA isolated from sorted CCR5-/CR2 cells. D21 and D84: genomic DNA samples isolated from sorted CCR5-/CR2 cells at 21 and 84 days post transduction. B. T7EI analysis of nine potential off-target loci (SH2D5, ASB9P1, PRRT1, LINC00265, ENDOV, NR2F1, ASB9, CLPP and SOBP) to CR3 as well as on-target locus CCR5 control. “–”: genomic DNA isolated from parental TZM.bl; “+”: genomic DNA isolated from sorted CCR5-/CR3 cells. D21 and D84: genomic DNA samples isolated from sorted CCR5-/CR3 cells at 21 and 84 days post transduction.

To analyze potential off-target mutations, we first built the human exon region reference as described by Mali *et al*. [21]. Briefly, we downloaded a BED file from the UCSC Genome Browser (http://genome.ucsc.edu/cgi-bin/hgGateway). The file contains locations of coding regions of all RefSeq genes in the GRCh37/hg19 human genome. Using the merge Bed function of BED Tools we consolidated overlapping exon locations into merged exon sequence regions. We then downloaded all merged exon regions using the UCSC Table Browser. The downloaded sequence was used to build a bowtie reference database. The query sequences were mapped to the human exon region reference database with max mismatch setting of 0 (no mismatch) and 1 (a single mismatch) in the 3′ end 13 nucleotide seed and PAM (NGG) sequences of CR1, CR2 and CR3, respectively. We found that there are no exon sequences that are identical to or have a single mismatch with CR1 throughout all merged exon regions. However, there are 3 single mismatches (AKAP9, ULK1 and MED16) with CR2, and there are 9 single mismatches (SH2D5, ASB9P1, PRRT1, LINC00265, ENDOV, NR2F1, ASB9, CLPP and SOBP) with CR3 (S2 Table). We then designed 12 pairs of primers to amplify these potential off-target exon sequences (S2 Table). Using T7 endonuclease I assay, no indels were detected in the potential off-target sequences that are highly homologous to CR2 (Fig. 4A) or to CR3 (Fig. 4B) in stably transduced TZM.bl cells at 21 and 84 days post transduction as compared to genomic DNA isolated from mock-transduced TZM.bl cells.

### High efficiency of CCR5 gene disruption in human CD4^+^ T cells via lentiviral vectors expressing Cas9 and CR2

**Figure 5:**
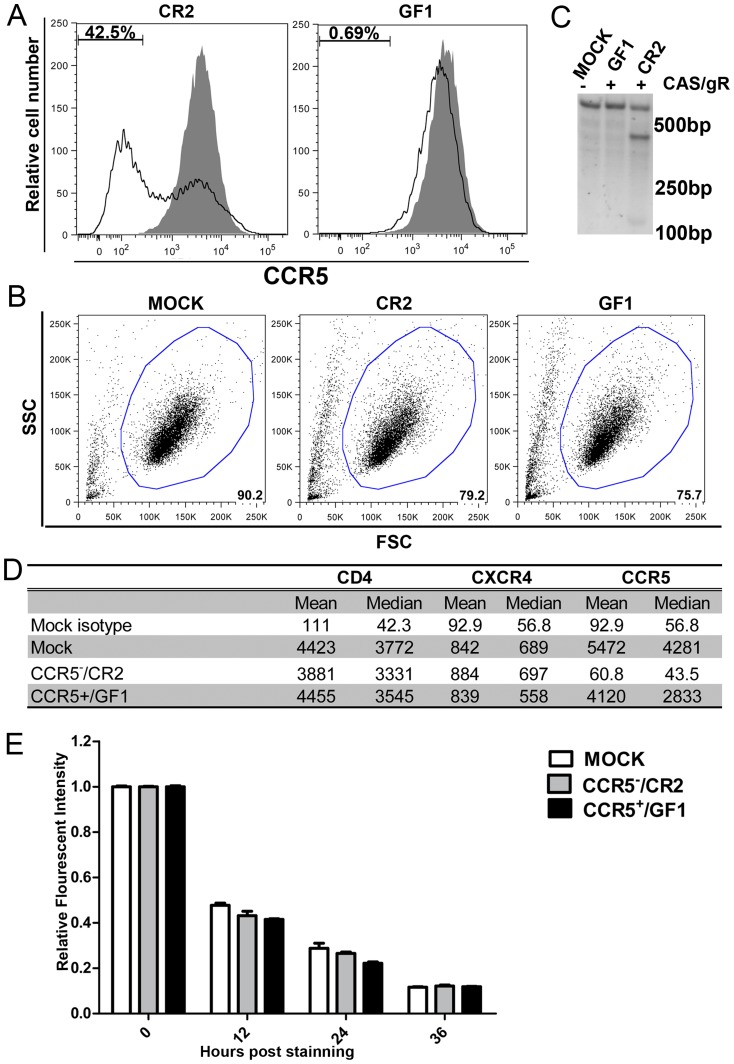
*CCR5* gene disruption by RNA-guided Cas9 endonuclease in transduced CEM ss-CCR5 cells. A. Cell surface expression of CCR5 on CEMss-CCR5 cells co-transduced with lentiviral vectors expressing Cas9-HA-NLS and one of sgRNA (CR2 or GF1) as compared to mock-transduced CEMss-CCR5 cells. B. FSC and SSC analysis mock-transduced CEMss-CCR5 cells and CEMss-CCR5 cells transduced with lentiviral vectors expressing Cas9-HA-NLS fusion protein and sgRNAs CR2 or GF1, respectively. Percentages of gated (live) cells are shown at the lower and right corner of the figures. C. *CCR5* gene disruption analysis in mock-transduced CEMss-CCR5 cells or CEMss-CCR5 cells co-transduced with lentiviral vectors expressing Cas9-HA-NLS and one of sgRNAs (CR2 or GF1) by T7EI assay. D. Mean and median values of fluorescence intensity of cell surface expression of CD4, CXCR4 and CCR5 in sorted CCR5-/CR2 and CCR5+/GF1 cells as compared to parental CEMss-CCR5 cells. E. Relative fluorescent intensity of DDAO-labeled CCR5-/CR2 and CCR5+/GF1 cells as compared to parental CEMss-CCR5 cells at 0, 12, 24 and 36 hours post culture.

While TZM.bl cells are widely used as target cells in HIV-1 neutralization assay, they are of epithelial origin [36]. Therefore, to further test *CCR5* gene editing, we co-transduced human CD4^+^ T cell line CEMss-CCR5 with lentiviral vectors expressing Cas9 and CR2 or GF1 control. Seven days after the transduction, cells were stained with anti-CCR5 or isotype control antibody followed by FACS analysis. Fig. 5A shows that 42.5% CCR5 negative cells was obtained in CEMss-CCR5 cells co-transduced with vectors expressing Cas9 and CR2, but no CCR5 reduction in cells co-transduced with vectors expressing Cas9 and GF1 control. FSC and SSC analysis shows that 75.7 and 79.2% of CEMss-CCR5 cells transduced with lentiviral vectors expressing Cas9-HA-NLS fusion protein and sgRNAs CR2 or GF1 were live cells, which was slightly lower than mock-transduced cells (Fig. 5B). Fig. 5C shows that *CCR5* gene was effectively disrupted in CEMss-CCR5 cells co-transduced with vectors expressing Cas9 and CR2, but not in cells vectors expressing Cas9 and GF1 control, using T7 endonuclease I assay.

We next sorted CCR5 negative cell populations in CEMss-CCR5 cells co-transduced with lentiviral vectors expressing Cas9 and CR2 by FACS. After sorting, cell surface expression of CCR5, CXCR4 and CD4 were compared. While CCR5 expression was reduced in the CCR5^-^/CR2 cells, no significant difference in CD4 and CXCR4 expression (Fig. 5D) and in cell growth (Fig. 5E) was found in CCR5^-^/CR2 cells as compared to mock-transduced and CCR5^+^/GF1 cells.

### Disruption of CCR5 in human CD4^+^ T cells confers resistance to R5-tropic viruses and a selective advantage during R5-tropic HIV-1 infection

**Figure 6:**
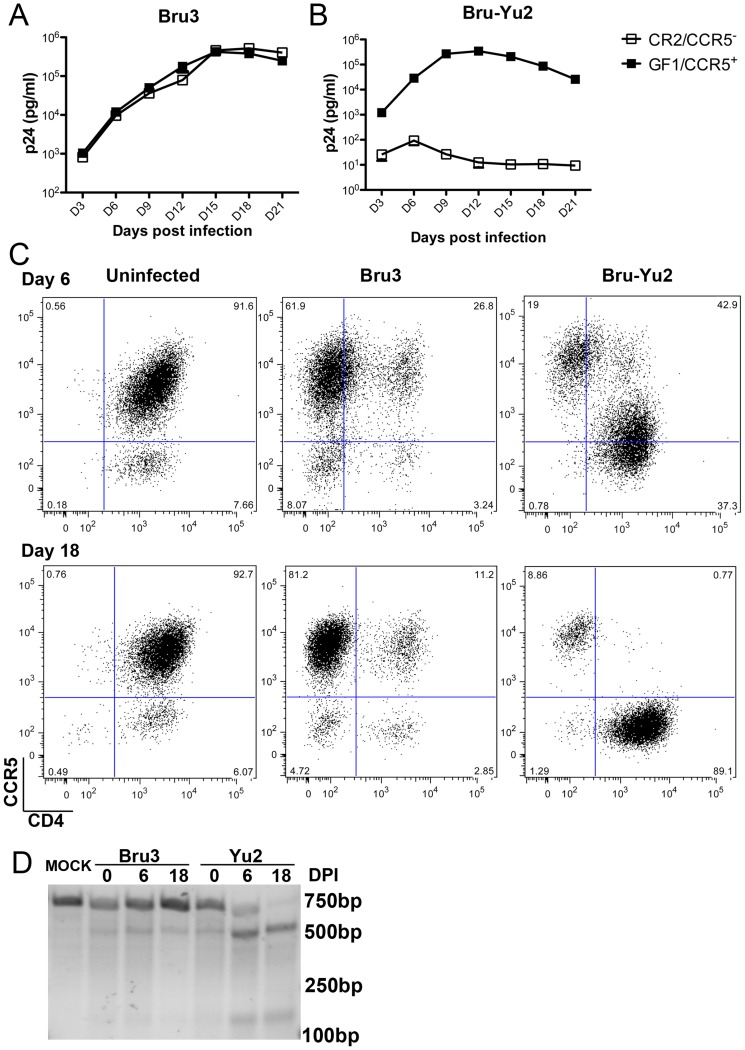
CCR5-/CR2 cells confer resistant to R5-tropic viruses and exhibits selective advantage during R5-tropic HIV-1 infection. A. HIV-1 replication in sorted CCR5-/CR2 and CCR5+/GF1 cells infected with X4-tropic HIV-1 Bru3. B. HIV-1 replication in sorted CCR5-/CR2 and CCR5+/GF1 cells infected with R5-tropic HIV-1 Bru-Yu2. C. FACS analysis of cell surface expression of CD4 and CCR5 in mixtures of CCR5-/CR2 and parental CEMss-CCR5 cells at 6 and 18 days post-infection with X4-tropic HIV-1 Bru3 (middle panel), R5-tropic HIV-1 Bru-Yu2 (right panel), or no virus control (left panel). D. *CCR5* gene analysis on uncleaved versus cleaved bands in parental CEMss-CCR5 cells (line 1) or CCR5-/CR2 and parental CEMss-CCR5 mixture at 0, 6 and 18 days post infection with X4 tropic HIV-1 Bru3 (lines 2 to 4) or R5-tropic HIV-1 Bru-Yu2 (lines 5 to 7) by T7EI assay. DPI: days post infection.

CCR5^-^/CR2 and CCR5^+^/GF1 cells were then infected with either X4-tropic HIV-1 Bru-3 or R5-tropic HIV-1 Bru-Yu2. After the challenge, cells and culture supernatants were collected every 3 days and replenished with fresh medium for a total of 21 days. As measured by HIV-1 gag p24 in the culture supernatants, similar viral replication was observed when CCR5^-^/CR2 and CCR5^+^/GF1 cells were infected with X4-tropic HIV-1 Bru3 (Fig. 6A). However, a significant reduction in HIV-1 replication was found in CCR5^-^/CR2 cells as compared to CCR5^+^/GF1 cells when cells were infected with the R5-tropic HIV-1 Bru-Yu2 (Fig. 6B).

To test whether *CCR5* gene-disrupted cells had a selective advantage over CCR5 expressing cells during R5-tropic HIV-1 infection, we mixed CCR5^-^/CR2 and mock-transduced CEMss-CCR5 cells at 1∶9 ratio and then challenged cell mixture with either X4-tropic HIV-1 Bru-3 or R5-tropic HIV-1 Bru-Yu2. After the challenge, cells and culture supernatants were collected every 3 days and replenished with fresh medium for a total of 18 days. At day 6 and 18 post-infection, portions of the cultured cells were stained with anti-CCR5 and anti-CD4 antibodies followed by FACS analysis, and genomic DNA were extracted from another portion of cells and subjected to T7 endonuclease I assay. Fig. 6C shows that the 1∶9 ratio of CCR5^-^ and CCR5^+^ cells at both day 6 and 18 post-infection with the X4-tropic HIV-1 Bru3 was maintained (middle panel), similar to uninfected cell mixture (left panel), although infection resulted in down regulation of CD4. By contrast, in the cell mixture infected with R5-tropic HIV-1 Bru-Yu2, the ratio of CCR5^-^ and CCR5^+^ cells was significantly altered. At the 6 days post-infection the ratio of CCR5^-^ and CCR5^+^ cells became 4∶6 (upper/right panel) and at the 18 days post-infection the ratio became 9∶1 (lower/right panel). Similar results were also found when we analyzed the ratios of cleaved versus uncleaved CCR5 bands at days 6 and 18 post-infection in the cell mixtures infected with X4-tropic HIV-1 Bru3 or R5-tropic HIV-1 Bru-Yu2 using T7 endonuclease I assay (Fig. 6D). Thus, taken together we concluded that *CCR5* gene-disrupted cells are resistant to R5 viruses and exhibit a selective survival advantage during R5 HIV-1 infection.

### High efficiency of CCR5 gene disruption in human CD4^+^ T cells via a single lentiviral vector expressing both Cas9 and CR2

**Figure 7:**
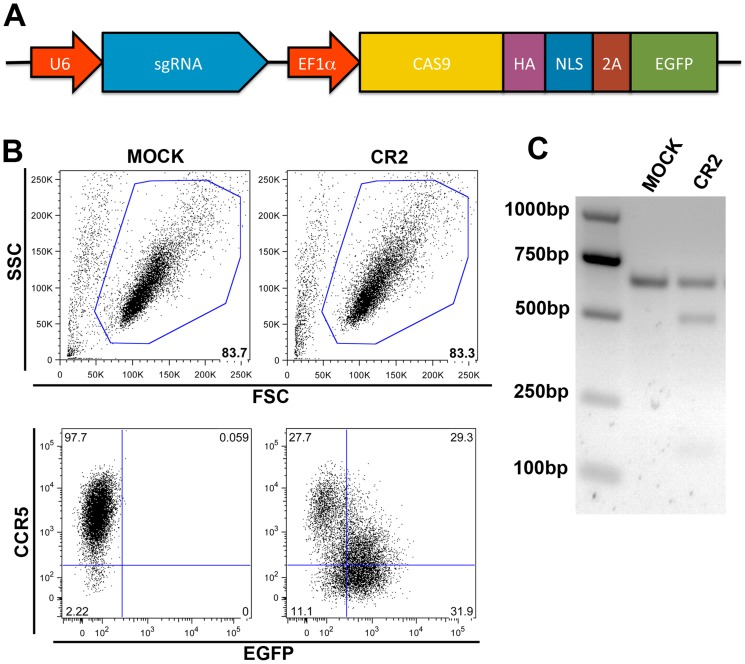
*CCR5* gene disruption in CEMss-CCR5 cells transduced with a single lentiviral vector expressing both CR2 sgRNA and Cas9. A. Schematic diagrams of a lentiviral transfer vector pRRL-CR2 sgRNA-Cas9/EGFP. Cas9: Cas9 endonuclease; CR2 sgRNA: single guided RNA CR2; HA: HA epitope tag; NLS: nuclear localization signal; U6: polymerase III U6 promoter sequence; EF1α: promoter sequence derived from elongation factor 1α; 2A: 2A self cleaving peptide sequence; and EGFP: sequence encoding enhanced green fluorescent protein. B. FSC and SSC analysis in mock-transduced CEMss-CCR5 cells and CEMss-CCR5 cells transduced with a single lentiviral vector pRRL-CR2 sgRNA-Cas9/EGFP. Percentages of gated (live) cells are shown at the lower and right corner of the top-right and top-left panels. CCR5 and EGFP analysis in mock-transduced CEMss-CCR5 cells and CEMss-CCR5 cells transduced with a single lentiviral vector pRRL-CR2 sgRNA-Cas9/EGFP. C. *CCR5* gene disruption analysis in mock-transduced CEMss-CCR5 cells or CEMss-CCR5 cells transduced with a single lentiviral vector pRRL-CR2 sgRNA-Cas9/EGFP by T7EI assay.

Having demonstrated that co-transducing CEMss-CCR5 with two individual lentiviral vectors expressing Cas9 and CR2 efficiently disrupt *CCR5* gene, we next constructed a single lentiviral transfer vector expressing both CR2 and Cas9 along with EGFP reporter gene (Fig. 7A). In this single transfer vector CR2 was driven by U6 promoter and Cas9/HA/NLS/2A/EGFP fusion gene was driven by elongation factor-1α (EF1α) promoter. Seven days after the transduction, cells were stained with anti-CCR5 or isotype control antibody followed by FACS analysis. Fig. 7B shows that 43 (11.1 + 31.9)% CCR5 negative cells was obtained in CEMss-CCR5 cells transduced with this single vector (the bottom-left panel); whereas only 2.2 (2.2+0)% CCR5 negative cells was observed in mock-transduced cells (the bottom-right panel). Fig. 7C shows that *CCR5* gene was effectively disrupted in CEMss-CCR5 cells transduced with this single vector using T7 endonuclease I assay. CCR5 negative cells (43%) obtained in CEMss-CCR5 cells transduced with this single vector co-expressing sgRNA (CR2) and CAS9 was similar to 42.5% CCR5 negative cells transduced with two vectors that express CR2 and CAS9 separatedly (Fig. 5A). Certainly, there are several ways to further improve the efficiency of CCR5 gene disruption, such as higher MOI used in transduction, multiple rounds of transduction and test more sgRNAs.

## Discussion

Although the first reported “cure” of HIV-1 infection came from a CCR5Δ32 homozygous allogeneic bone marrow transplantation [52], [53], few individuals are likely to benefit from this treatment due to toxicity of allogeneic rejection and limitation of HLA-matched CCR5Δ32 homozygous donors. To overcome these problems, a CCR5-ZFN has been developed to inactivate the *CCR5* gene [6], [11]–[15]. *CCR5* gene disruption was observed following a single round of transduction with adenovirus vectors expressing the CCR5-ZFN or electroporation of a plasmid DNA expressing CCR5-ZFN [11], [13]. Moreover, significant increases in *CCR5* gene-disrupted cells and lower plasma viremia were observed in NOG mice after challenged with HIV-1 [11]. Currently CCR5-ZFN-transduced autologous T cells are being tested in two Phase I clinical trials [10], [16]. In the present study, we tested whether lentiviral vectors expressing the Cas9 and sgRNAs of *CCR5* could be used to engineer HIV-1 resistance.

Perhaps, the most important findings of this study are that a single round of co-transduction of human CD4^+^ cells with the lentiviral vectors expressing Cas9 and sgRNAs CR1, CR2 or CR3 or a single round of transduction of human CD4^+^ cells with a single lentiviral vector expressing both CR2 and Cas9 resulted in high efficiency of human *CCR5* gene disruption in these cells (Fig. 1B and C; Fig. 2; Fig. 5 and Fig. 7). The *CCR5* gene-modified cells are highly resistant to R5-tropic HIV-1 infection including transmitted founder (T/F) viruses (Fig. 3 and 6A and B) and exhibit a selective survival advantage over cells with unmodified *CCR5* during R5-tropic HIV-1 infection (Fig. 6C and D). These findings indicate that the efficiency of *CCR5* gene disruption by the Cas9 and sgRNA system is at least the same as, if it is not higher than, those by CCR5-ZFN or CCR5-TALEN [11], [15]. However, further side-by-side study is needed to compare CCR5 gene disruption by these three gene editing approaches.

Another important finding is that we did not detect off-target mutations in 12 loci that contain one nucleotide mismatch to the 3′ 13 nucleotide seed regions and PAM (NGG) of CR2 or CR3 in cells co-transduced with lentiviral vectors expressing Cas9 and CR2 or CR3 using T7 endonuclease I assay even at 84 days post transduction (Fig. 4), indicating that likely co-transducing cells with lentiviral vectors expressing Cas9 and CR2 or CR3 does not induce mutations at potential off-target sites that are highly homologous to CR2 or CR3 in HIV-1 susceptible human CD4^+^ cells. However, due to its lower sensitivity, the T7 endonuclease I assay can only detect off-target mutations that occur at frequencies equal to or high than 1% [54]. Thus, to completely rule out potential mutations in these 12 loci that contain one nucleotide mismatch to the 3′ 13 nucleotide seed regions and PAM (NGG) of CR2 or CR3, it would be necessary to perform deep sequencing to thoroughly rule out much lower frequencies of mutations. Using deep sequencing Cho *et al.* recently showed high frequencies of CCR5 indels in K562 cells co-transfected with DNA plasmids encoding Cas9 and 2 of 28 CCR5 sgRNAs, but no mutations at measurable frequency (about 0.01%) at these putative off-target sites to these 2 CCR5 sgRNAs [33]. Interestingly, when testing for potential off-target mutations in the *CCR2* gene with an additional 9 CCR5 sgRNAs, one of the CCR5 sgRNAs (off#3), which has an identical crRNA sequence of our CR2, did not cause *CCR2* gene mutations that were identifiable by deep sequencing (Figure 2 in ref 33). Thus, taken together the findings by Cho *et al* [33] and by our present study indicate a low possibility for off-target mutations induced by Cas9 and CR2.

In this study, we used lentiviral vectors to deliver Cas9 and sgRNAs into HIV-1 susceptible human CD4^+^ cells. Therefore, the high level of *CCR5* gene disruption observed in our study could be due to efficient gene delivery by lentiviral vectors to these cells. Interestingly, we observed that although *CCR5* gene disruption could be detected at 3 days post transduction, the disruption continued increasing until 9 days post transduction (Wang *et al.* data not shown). Similar observation was also reported in other genes disrupted by lentiviral vectors expressing Cas9 and sgRNAs [55]. However, since lentiviral vectors are integrated into host cell genome and Cas9 and sgRNAs are likely to be persistently expressed, the long-term effect of vector integration and persistent expression of Cas9 and sgRNAs on the target cell genome is currently unknown. To overcome this potential adverse effects, a integrase defective lentiviral vector that co-expresses Cas9 and sgRNAs should be tested. Otherwise, similar to vectors reported by Levine *et al* [56], the lentiviral vectors used in this study to express Cas9 and CCR5 sgRNAs may have the potential advantage of *in vivo* vector mobilization when they are used to treat HIV-1 patients.

After successfully disrupting *CCR5* gene in human T cell lines, we have made several attempts to disrupt *CCR5* gene in human primary T cells from multiple donors with lentiviral vectors expressing Cas9 and sgRNA CR2, including a single lentiviral transfer construct that co-expresses Cas9 and sgRNA CR2 along with GFP marker protein. Prior to transduction, we have also tried several stimulation protocols such as Phytohemagglutinin (PHA), anti-CD3 and anti-CD28 antibody-coated plate, or anti-CD3 and anti-CD28 antibody-coated beads. Without exception, all our attempts yielded negative results, i.e. no *CCR5* gene disruption could be detected by T7 endonuclease I assay, although all these constructs effectively disrupt *CCR5* gene in human T cell lines and decent transduction efficency of these constructs into the primary T cells was obtained and Cas9 RNA transcripts were detected by real-time PCR (Wang et al. data not shown). Similarly, when we generated single lentiviral transfer constructs to disrupt *CD4* and *CXCR4* genes, we observed the same results, i.e. the single lentiviral vectors efficiently disrupt *CD4* and *CXCR4* genes in transduced human T cell line, but not in primary human T cells (Wang et al. Data not shown). At this time, we do not know what causes such a dramatic difference in *CCR5, CD4* and *CXCR4* gene disruption between T cell lines and primary T cells. But our unexpected results in primary T cells serve as a cautionary warning for researchers like us who are interested in developing the CRISPR/CAs9 system into clinical applications.

In summary, we tested *CCR5* gene disruption in HIV-1 susceptible cells using lentiviral vectors expressing Cas9 and sgRNAs CR1, CR2 or CR3. We demonstrated that co-transducing lentiviral vectors expressing Cas9 and CR2 yields high frequencies of on-target (*CCR5*), but not off-target, genome modification in a HIV-1 susceptible human CD4^+^ cell lines. *CCR5* gene-disrupted cells are highly resistant to R5-tropic HIV-1 infection and exhibit selective advantage over *CCR5* gene-undisrupted cells during R5-tropic HIV-1 infection. Finally, we showed that transducing human CD4 T cells with a single lentiviral vector expressing both sgRNA CR2 and Cas9 efficiently disrupt *CCR5* gene. Thus, this lentiviral vector expressing both Cas9 and sgRNA CR2 may be a viable alternative to CCR5-ZFN gene editing, i.e. to recapitulate the success of CCR5Δ32 homozygous allogeneic transplantation via CCR5 gene-disruption of autologous hematopoietic stem cells and CD4^+^ T cells. However, since all our attempts to disrupt *CCR5* gene in human primary T cells with lentiviral vectors expressing Cas9 and sgRNA CR2 yielded negative results, finding out why lentiviral vectors expressing Cas9 and sgRNA CR2 fail to disrupt *CCR5* gene in primary T cells and how efficient lentiviral vectors expressing Cas9 and sgRNA CR2 disrupt *CCR5* gene in human hematopoietic stem cells will be extremely important for moving these Cas9 and sgRNA CR2 contructs into clinical settings.

## Supporting Information

S1 Fig **DNA sequences of CAS9 and sgRNAs.** A. DNA sequence of *CAS9*. Green color represents the sequence of HA tag and red color represents the sequence of the nucleus localization signal of SV40. B. DNA sequence of U6 promoter and sgRNAs. Gray color represents sequence of U6 promoter, green color represents the sequence of target site and blue color represents the scaffold RNAs. The four sgRNA sequences are listed in the Table.
(PDF)

S2 Fig **Sanger sequencing of** ***CCR5*** **gene target sites by CR1, CR2 or CR3 in co-transduced TZM.bl cells.**
(PDF)

S1 Table **Primer pairs used to amplify** ***CCR5*** **gene target sites for T7EI assay and Sanger sequencing.**
(PDF)

S2 Table **The list of potential off- target sites that are highly homologous to CR2 or CR3 and prime pairs to amplify these potential off-target sites for T7EI assay.**
(PDF)
