# Supplementary material for: CCR5 Gene Disruption via Lentiviral Vectors Expressing Cas9 and Single Guided RNA Renders Cells Resistant to HIV-1 Infection
Source: PLoS One. 2014 Dec 26;9(12):e115987. doi: 10.1371/journal.pone.0115987 (PMC4277423; doi:10.1371/journal.pone.0115987)
Supplement: S1 Fig — DNA sequences of CAS9 and sgRNAs. A. DNA sequence of CAS9. Green color represents the sequence of HA tag and red color represents the sequence of the nucleus localization signal of SV40. B. DNA sequence of U6 promoter and sgRNAs. Gray color represents sequence of U6 promoter, green color represents the sequence of target site and blue color represents the scaffold RNAs. The four sgRNA sequences are listed in the Table. (PDF) [file pone.0115987.s001.pdf]

A

CAS9-HA-NLS DNA sequence

ggatccaccatggacaagaagtacagcatcggcctggacatcggcaccaacagcgtgg
gctgggccgtgatcaccgacgagtacaaggtgcccagcaagaagttcaaggtgctgggc
aacaccgaccgccacagcatcaagaagaacctgatcggcgccctgctgttcgacagcg
gcgagaccgccgaggccacccgcctgaagcgcaccgcccgccgccgctacacccgcc
gcaagaaccgcatctgctacctgcaggagatcttcagcaacgagatggccaaggtggac
gacagcttcttccaccgcctggaggagagcttcctggtggaggaggacaagaagcacga
gcgccaccccatcttcggcaacatcgtggacgaggtggcctaccacgagaagtacccca
ccatctaccacctgcgcaagaagctggtggacagcaccgacaaggccgacctgcgcctg
atctacctggccctggcccacatgatcaagttccgcggccacttcctgatcgagggcgacct
gaaccccgacaacagcgacgtggacaagctgttcatccagctggtgcagacctacaacc
agctgttcgaggagaaccccatcaacgccagcggcgtggacgccaaggccatcctgag
cgcccgcctgagcaagagccgccgcctggagaacctgatcgcccagctgcccggcgag
aagaagaacggcctgttcggcaacctgatcgccctgagcctgggcctgacccccaacttc
aagagcaacttcgacctggccgaggacgccaagctgcagctgagcaaggacacctacg
acgacgacctggacaacctgctggcccagatcggcgaccagtacgccgacctgttcctgg
ccgccaagaacctgagcgacgccatcctgctgagcgacatcctgcgcgtgaacaccgag
atcaccaaggcccccctgagcgccagcatgatcaagcgctacgacgagcaccaccagg
acctgaccctgctgaaggccctggtgcgccagcagctgcccgagaagtacaaggagatc
ttcttcgaccagagcaagaacggctacgccggctacatcgacggcggcgccagccagg
aggagttctacaagttcatcaagcccatcctggagaagatggacggcaccgaggagctg
ctggtgaagctgaaccgcgaggacctgctgcgcaagcagcgcaccttcgacaacggca
gcatccccccaccagatccacctgggcgagctgcacgccatcctgcgccgccaggagga
cttctaccccttcctgaaggacaaccgcgagaagatcgagaagatcctgaccttccgcatc
ccctactacgtgggccccctggcccgcggcaacagccgcttcgcctggatgacccgcaa
gagcgaggagaccatcaccccctggaacttcgaggaggtggtggacaagggcgccag
cgcccagagcttcatcgagcgcatgaccaacttcgacaagaacctgcccaacgagaag
gtgctgcccaagcacagcctgctgtacgagtacttcaccgtgtacaacgagctgaccaag
gtgaagtacgtgaccgagggcatgcgcaagcccgccttcctgagcggcgagcagaaga
aggccatcgtggacctgctgttcaagaccaaccgcaaggtgaccgtgaagcagctgaag
gaggactacttcaagaagatcgagtgcttcgacagcgtggagatcagcggcgtggagga
ccgcttcaacgccagcctgggcacctaccacgacctgctgaagatcatcaaggacaagg
acttcctggacaacgaggagaacgaggacatcctggaggacatcgtgctgaccctgacc
ctgttcgaggaccgcgagatgatcgaggagcgcctgaagacctacgcccacctgttcgac
gacaaggtgatgaagcagctgaagcgccgccgctacaccggctggggccgcctgagcc
gcaagctgatcaacggcatccgcgacaagcagagcggcaagaccatcctggacttcctg
aagagcgacggcttcgccaaccgcaacttcatgcagctgatccacgacgacagcctgac
cttcaaggaggacatccagaaggcccaggtgagcggccagggcgacagcctgcacga
gcacatcgccaacctggccggcagccccgccatcaagaagggcatcctgcagaccgtg
aaggtggtggacgagctggtgaaggtgatgggccgccacaagcccgagaacatcgtgat
cgagatggcccgcgagaaccagaccacccagaagggccagaagaacagccgcgag
cgcatgaagcgcatcgaggagggcatcaaggagctgggcagccagatcctgaaggag

caccccgtggagaacacccagctgcagaacgagaagctgtacctgtactacctgcagaa
cggccgcgacatgtacgtggaccaggagctggacatcaaccgcctgagcgactacgac
gtggaccacatcgtgccccagagcttcctgaaggacgacagcatcgacaacaaggtgct
gacccgcagcgacaagaaccgcggcaagagcgacaacgtgcccagcgaggaggtgg
tgaagaagatgaagaactactggcgccagctgctgaacgccaagctgatcacccagcgc
aagttcgacaacctgaccaaggccgagcgcggcggcctgagcgagctggacaaggcc
ggcttcatcaagcgccagctggtggagacccgccagatcaccaagcacgtggcccagat
cctggacagccgcatgaacaccaagtacgacgagaacgacaagctgatccgcgaggtg
aaggtgatcaccctgaagagcaagctggtgagcgacttccgcaaggacttccagttctac
aaggtgcgcgagatcaacaactaccaccacgcccacgacgcctacctgaacgccgtggt
gggcaccgccctgatcaagaagtaccccaagctggagagcgagttcgtgtacggcgact
acaaggtgtacgacgtgcgcaagatgatcgccaagagcgagcaggagatcggcaagg
ccaccgccaagtacttcttctacagcaacatcatgaacttcttcaagaccgagatcaccctg
gccaacggcgagatccgcaagcgcccccctgatcgagaccaacggcgagaccggcga
gatcgtgtgggacaagggccgcgacttcgccaccgtgcgcaaggtgctgagcatgcccc
aggtgaacatcgtgaagaagaccgaggtgcagaccggcggcttcagcaaggagagcat
cctgcccaagcgcaacagcgacaagctgatcgcccgcaagaaggactgggaccccaa
gaagtacggcggcttcgacagccccaccgtggcctacagcgtgctggtggtggccaaggt
ggagaagggcaagagcaagaagctgaagagcgtgaaggagctgctgggcatcaccat
catggagcgcagcagcttcgagaagaaccccatcgacttcctggaggccaagggctaca
aggaggtgaagaaggacctgatcatcaagctgcccaagtacagcctgttcgagctggag
aacggccgcaagcgcatgctggccagcgccggcgagctgcagaagggcaacgagctg
gccctgcccagcaagtacgtgaacttcctgtacctggccagccactacgagaagctgaag
ggcagccccgaggacaacgagcagaagcagctgttcgtggagcagcacaagcactac
ctggacgagatcatcgagcagatcagcgagttcagcaagcgcgtgatcctggccgacgc
caacctggacaaggtgctgagcgcctacaacaagcaccgcgacaagcccatccgcga
gcaggccgagaacatcatccacctgttcaccctgaccaacctgggcgcccccgccgcctt
caagtacttcgacaccaccatcgaccgcaagcgctacaccagcaccaaggaggtgctg
gacgccaccctgatccaccagagcatcaccggcctgtacgagacccgcatcgacctgag
ccagctgggcggcgactacccctacgacgtgcccgactacgccacccccccaagaag
cgcaaggtgtaagtcgac
HA
NLS

B

U6 promoter + target RNA + guide RNA scaffold:
TGTACAAAAAAGCAGGCTTTAAAGGAACCAATTCAGTCGACTG
GATCCGGTACCAAGGTCGGGCAGGAAGAGGGCCTATTTCCC
ATGATTCCTTCATATTTGCATATACGATACAAGGCTGTTAGAG
AGATAATTAGAATTAATTTGACTGTAAACACAAAGATATTAGTA
CAAAATACGTGACGTAGAAAGTAATAATTTCTTGGGTAGTTTG
CAGTTTTAAAATTATGTTTTAAAATGGACTATCATATGCTTACC
GTAACTTGAAAGTATTTCGATTTCTTGGCTTTATATATCTTGTG

GAAAGGACGAAACACC**GNNNNNNNNNNNNNNNNNNNGTTTT**
AGAGCTAGAAATAGCAAGTTAAAATAAGGCTAGTCCGTTAT
CAACTTGAAAAAGTGGCACCGAGTCGGTGCTTTTTTT

sgRNA	Strand polarity	Target sequence
CR1	+	GCTTGTGACACGGACTCAAG
CR2	+	GGTCCTGCCGCTGCTTGTCA
CR3	-	GTAAACTGAGCTTGCTCGCT
GF1	+	GGAGCGCACCATCTTCTTCA
