# Supplementary material for: CCR5 Gene Disruption via Lentiviral Vectors Expressing Cas9 and Single Guided RNA Renders Cells Resistant to HIV-1 Infection
Source: PLoS One. 2014 Dec 26;9(12):e115987. doi: 10.1371/journal.pone.0115987 (PMC4277423; doi:10.1371/journal.pone.0115987)
Supplement: S2 Fig — Sanger sequencing of CCR5 gene target sites by CR1, CR2 or CR3 in co-transduced TZM.bl cells. (PDF) [file pone.0115987.s002.pdf]

CR1 Wild-type
GCTCCCGAGCGAGCAAGCTCAGTTTACACCCGATCCACTGGGGAGCAGGAAATATCTGTGGGCTTGTGACACGGACTCAAGTGGGCTGGTGACCCAGTCAGAGTTGTGCACATGGCTTAGTTTT WT
CR1 genome and transgene targeting deletions (19)
GCTCCCGAGCGAGCAAGCTCAGTTTACACCCGATCCACTGGGGAGCAGGAAATATCTGTGGGCTTGTGACACGGA--CAAGTGGGCTGGTGACTCAGTCAGAGTTGTGCACATGGCTTAGTTTT -2
GCTCCCGAGCGAGCAAGCTCAGCTTACACCCGATCCACTGGGGAGCAGGAAATATCTGTGGGCTTGTGACACGGA----AGTGGGCTGGTGACCCAGTCAGAGTTGTGCACATGGCTTAGTTTT -4
GCTCCCGAGCGAGCAAGCTCAGTTTACACCCGATCCACTGGGGAGCAGGAAATATCTGTGGGCTTGTGTAA--------AGTGGGCTGGTGACCCAGTCAGAGTTGTGCACATGGCTTAGTTTT -8
GCTCCCGAGCGAGCAAGCTCAGTTTACACCCGATCCACTGGGGAGCAGGAAATATCTGTGGGCTTGTGACACGGA----------CTGGTGACCCAGTCAGAGTTGTGCACATGGCTTAGTTTT *-10*
GCTCCCGAGCGAGCAAGCTCAGTTTACACCCGATCCACTGGGGAGCAGGAAATATCTGTGGGCTTGTG----------------ACTGGTGACCCAGTCAGAGTTGTGCACATGGCTTAGTTTT -16
GCTCCCGAGCGAGCAAGCTCAGTTTACACCCGATCCACTGGGGAGCAGGAAATATCTGTGGGCTTGTGACACGGACT-----------------CAGTCAGAGTTGTGCACATGGCTTAGTTTT -17 ×2
GCTCCCGAGCGAGCAAGCTCAGTTTACACCCGATCCACTGGGGAGCAGGAAATATCTGTGGGCTGGTG-----------------------ACCCAGTCAGAGTTGTGCACATGGCTTAGTTTT *-23* ×2
GCTACCGAGCGAGCAAGCTCAGTTTACACCCGATCCACTGGGGAGCAGGAAATATCTGTGGGCTTGTG-----------------------ACCCAGTCAGAGTTGTGCACATGGCTTAGTTTT **-23** ×4
GCTCCCGAGCGAGCAAGCTCAGTTTACACCCGATCCACTGGGGAG--TTCTTAGTTTT -68
GCTCCCTAGCACG--TTT -108 ×5
CR1 genome targeting deletions (15)
GCTCCCGAGCGAGCAAGCTCAGTTTACACCCGATCCACTGGGGAGCAGGAAATATCTGTGGGCTTGTGACACGGACTG--GTGGGCTGGTGACCCAGTCAGAGTTGTGCACATGGCTTAGTTTT -2
GCTACCGAGCGAGCAAGCTCAGTTTACACCCGATCCACTGGGGAGCAGGAAATATCTGTGGGCTTGTGACACGGACTC----GGGCTGGTGACCCAGTCAGAGTTGTGCACATGGCTTAGTTTT -4
GCTCCCGAGCGAGCAAGCTCAGTTTACACCCGATCCACTGGGGAGCAGGAAATATCTGTGGGCTTGTGACACGGACT----------GGTGACCCAGTCAGAGTTGTGCACATGGCTTAGTTTT *-10*
GCTCCCGAGCGAGCAAGCTCAGTTTACACCCGATCCACTGGGGAGCAGGAAATATCTGTGGACTGGTGAC-----------------------CCAGTCAGAGTTGTGCACATGGCTTAGTTTT -23
GCTACCGAGCGAGCAAGCTCAGTTTACACCCGATCCACTGGGGAGCAGGAAATATCTGTGGGCTGGTGAC-----------------------CCAGTCAGAGTTGTGCACATGGCTTAGTTTT *-23* ×6
GCTACCGAGCGAGCAAGCTCAGTTTACACCCGATCCACTGGGGAGCAGGAAATATCTGTGGGCTTGTGAC-----------------------CCAGTCAGAGTTGTGCACATGGCTTAGTTTT **-23** ×2
GCTACCGAGCGAGCAAGCTCAGTTTACACCCGATCCACTGGGGAGCAGGAAATATCTGTGGGCTTGTG--CACATGGCTTAGTTTT -40
GCT--GGTGACCCAGTCAGAGTTGTGCACATGGCTTAGTTTT -84
TTGGTTTTGTGGGCAACATGCTGGTCATCCTCATCCTGATAAACTGCAAAAGGCTGAAGAGCATGACTGACATCTACCTGCTCAACCTGGCCATCTCTGACCTGTTTTTCCA-----------
----

-- -839

---GGCTGGTGACCCAGTCAGAGTTGTGCACATGGCTTAGTTTT

CR2 Wild-type
GGGGCTGGTCCTGCCGCTGCTTG--TCATGGTCATCTGCTACTCGGGAATCCTAAAAACTCTGCTTCGGTGTCGAAATGAGAAGAAGAGGCACAGGCTGTGAGGCTTATCTTCACCATCATG WT
CR2N genome and transgene targeting deletions (8)
GGGGCTGGTCCTGCCGCTGCT-------TGGTCATCTGCTACTCGGGAATCCTAAAAACTCTGCTTCGGTGTCGAAATGAGAAGAAGAGGCACAGGCTGTGAGGCTTATCTTCACCATCATG *-5*
GGGGCTGGTCCTGCCGCT-------------------GCTACTCGGGAATCCTAAAAACTCTGCTTCGGTGTCGAAATGAGAAGAAGAGGCACAGGCTGTGAGGCTTATCTTCACCATCATG -17 ×4
GGGGCTGGTCCTGCCGCT----------------------ACTCGGGAATCCTAAAAACTCTGCTTCGGTGTCGAAATGAGAAGAAGAGGCACAGGCTGTGAGGCTTATCTTCACCATCATG -20
GGG-----------------------CATGGTCATCTGCTACTCGGGAATCCTAAAAACTCTGCTTCGGTGTCGAAATGAGAAGAAGAGGCACAGGCTGTGAGGCTTATCTTCACCATCATG -21
GGGGCTGGTCCTGCCGCT--------------------------GGGAATCCTAAAAACTCTGCTTCGGTGTCGAAATGAGAAGAAGAGGCACAGGCTGTGAGGCTTATCTTCACCATCATG -24
CR2 genome and transgene targeting insertion (1)
GGGGCTGGTCCTGCCGCTGCTTGC-TCATGGTCATCTGCTACTCGGGAATCCTAAAAACTCTGCTTCGGTGTCGAAATGAGAAGAAGAGGCACAGGCTGTGAGGCTTATCTTCACCATCATG 1
CR2 genome targeting deletions (16)
GGGGCTGGTCCTGCCGCTG-------CATGGTCATCTGCTACTCGGGAATCCTAAAAACTCTGCTTCGGTGTCGAAATGAGAAGAAGAGGCACAGGCTGTGAGGCTTATCTTCACCATCATG -5
GGGGCTGGTCCTGCCGCTGCT-------TGGTCATCTGCTACTCGGGAATCCTAAAAACTCTGCTTCGGTGTCGAAATGAGAAGAAGAGGCACAGGCTGTGAGGCTTATCTTCACCATCATG *-5*
GGGGCTGGTCCTGCCGC----------ATGGTCATCTGCTACTCGGGAATCCTAAAAACTCTGCTTCGGTGTCGAAATGAGAAGAAGAGGCACAGGCTGTGAGGCTTATCTTCACCATCATG -8
GGGGCTGGTCCTGCCGCTG------------TCATCTGCTACTCGGGAATCCTAAAAACTCTGCTTCGGTGTCGAAATGAGAAGAAGAGGCACAGGCTGTGAGGCTTATCTTCACCATCATG -10
GGGGCTGGTCCTGCCGCTG-------------CATCTGCTACTCGGGAATCCTAAAAACTCTGCTTCGGTGTCGAAATGAGAAGAAGAGGCACAGGCTGTGAGGCTTATCTTCACCATCATG -11 ×2
GGGGCTGGTCCTG------------------TCATCTGCTACTCGGGAATCCTAAAAACTCTGCTTCGGTGTCGAAATGAGAAGAAGAGGCACAGGCTGTGAGGCTTATCTTCACCATCATG -16
GGGGCTGGTCCTGCCGCTG-------------------CTACTCGGGAATCCTAAAAACTCTGCTTCGGTGTCGAAATGAGAAGAAGAGGCACAGGCTGTGAGGCTTATCTTCACCATCATG -17 ×2
GGGGCTGGTCCTGCCGCTG----------------------CTTGGGAATCCTAAAAACTCTGCTTCGGTGTCGAAATGAGAAGAAGAGGCACAGGCTGTGAGGCTTATCTTCACCATCATG -20
GGGGTCGGTC-----------------------ATCTGCTACTCGGGAATCCTAAAAACTCTGCTTCGGTGTCGAAATGAGAAGAAGAGGCACAGGCTGTGAGGCTTATCTTCACCATCATG -21
GGGGCTGGTCCTGC-------------------------TACTCGGGAATCCTAAAAACTCTGCTTCGGTGTCGAAATGAGAAGAAGAGGCACAGGCTGTGAGGCTTATCTTCACCATCATG -23
GGGGCTGGTCCTGCCGCTGCTTG--------------------------------AAACTCTGCTTCGGTGTCGAAATGAGAAGAAGAGGCACAGGCTGTGAGGCTTATCTTCACCATCATG -30
GGGGCTGGTCCTGCCGCTG--CTTCGGTGTCGAAATGAGAAGAAGAGGCACAGGCTGTGAGGCTTATCTTCACCATCATG -42
GGGGCTGGTCCTGCCGCTGCTTGTT--CCATCATG -88
ACTCACTGGTGTTCATCTTTGGTTTTGTGGGCAACATGCTGGTCATCCTCATCCTGATAAACTGCAAAAGGCTGAAGAGCGTGACTGACATCTACCTGCTCAAC------------------
----
--- -426

--------------------------------------CTACTCGGGAATCCTAAAAACTCTGCTTCGGTGTCGAAATGAGAAGAAGAGGCACAGGCTGTGAGGCTTATCTTCACCATCATG
CR2 genome targeting insertion (1)
GGGGCTGGTCCTGCCGCTGCTTG-TTCATGGTCATCTGCTACTCGGGAATCCTAAAAACTCTGCTTCGGTGTCGAAATGAGAAGAAGAGGCACAGGCTGTGAGGCTTATCTTCACCATCATG 1
CR2 genome targeting deletion and insertion (2)
GGGGCTGGTCCTGCCGCTGCTTG-A-CATGG-CATCTGCTACTCGGGAATCCTAAAAACTCTGCTTCGGTGTCGAAATGAGAAGAAGAGGCACAGGCTGTGAGGCTTATCTTCACCATCATG (+1)/(-1)
GGGGCTGGTCCTGCCGCTGCT--CATCATGGTCATCTGCTACTCGGGAATCCTAAAAACTCTGCTTCGGTGTCGAAATGAGAAGAAGAGGCACAGGCTGTGAGGCTTATCTTCACCATCATG (+2)/(-2)

CR3 Wild-type
GTCACAAGCCCACAGATATTTCCTGCTCCCCAGTGGATCGGGTGTAAACT--GAGCTTGCTC--GCTCGGGAGCCTCTTGCTGGAAAATAGAACAGCATTTGCAGAAGCGTTTGGCAATGTGC WT
CR3 genome and transgene targeting deletions (13)
GTCACAAGCCCACAGATATTTCCTGCTCCCCAGTGGATCGGGTGTAAACT--GAGCTTGCTC----TCGGTAGCCTCTTGCTGGAAAATAGAACAGCATTTGCAGAAGCGTTTGGCAATGTGC -2
GTCACAAGCCCACAGATATTTCCTGCTCCCCAGTGGATCGGGTGTAAACT--GAGCTTGCTC------GGGAGCCTCTTGCTGGAAAATAGAACAGCATTTGCAGAAGCGTTTGGCAATGTGC -4 ×2
GTCACAAGCCCACAGATATTTCCTGCTCCCCAGTGGATCGGGTGTAAACT--GAGGTTG---------GGGAGCCTCTTGCTGGAAAATAGAACAGCATTTGCAGAAGCGTTTGGCAATGTGC -7
GTCACAAGCCCACAGATATTTCCTGCTCCCCAGTGGATCGGGTGTAAACT--GAGCT----------CGGGAGCCTCTTGCTGGAAAATAGAACAGCATTTGCAGAAGCGTTTGGCAATGTGC *-8* ×4
GTCACAAGCCCACAGATATTTCCTGCTCCCCAGTGGATCGGGTGTAAACT--GAGCTTGGT----------AGCCTCTTGCTGGAAAATAGAACAGCATTTGCAGAAGCGTTTGGCAATGTGC -8
GTCACAAGCCCACAGATATTTCCTGCTCCCCAGTGGATCGGGTGTAAACT--GAGCTTGCTC----------GCCTCTTGCTGGAAAATAGAACAGCATTTGCAGAAGCGTTTGGCAATGTGC -8
GTCACAAGCCCACAGATATTTCCTGCTCCCCAGTGGATCGGGTGTAAACT--GAGCTTG---------------------CTGGAAAATAGAACAGCATTTGCAGAAGCGCTTGGCAATGTGC *-19*
GTCACAAGCCCACAGATATTTCCTGCTCCCCAGTGGATC---------------------------GGGAGCCTCTTGCTGGAAAATAGAACAGCATTTGCAGAAGCGTTTGGCAATGTGC -25
GTCACAAGCCCACAGATATTTCCTGCTCCCCAGTGGATC--------------------------TCGGGAGCCTCTTGCTGGAAAATAGAACAGCATTTGCAGAAGCGTTTGGCAATGTGC -23
CR3 genome and transgene targeting insertion (6)
GTCACAAGCCCACAGATATTTCCTGCTCCCCAGTGGATCGGGTGTAAACT--GAGCTTGCTCT-GCTCGGGAGCCTCTTGCTGGAAAATAGAACAGCATTTGCAGAAGCGTTTGGCAATGTGC 1
GTCACGAGCCCACAGATATTTCCTGCTCCCCAGTGGATCGGGTGTAAACT--GAGCTTGCTCC-GCTCGGGAGCCTCTTGCTGAAAAATAGAACAGCATTTGCAGAAGCGTTTGGCAATGTGC 1 ×4
GTCACAAGCCCACAGATATTTCCTGCTCCCCAGTGGATCGGGTGTAAACT--GAGCTTGCTCG-GCTCGGGAGCCTCTTGCTGGAAAATAGAACAGCATTTGCAGAAGCGTTTGGCAATGTGC 1
CR3 genome targeting deletions (11)
GTCACAAGCCCACAGATATTTCCTGCTCCCCAGTGGATCGGGTGTAAACT--GAGCTTGCTC------GGGAGCCTCTTGCCGGAAAATAGAACAGCATTTGCAGAAGCGTTTGGCAATGTGC -4 ×3
GTCACAAGCCCACAGATATTTCCTGCTCCCCAGTGGATCGGGTGTAAACT--GAGCAAGCTCA------GTAGCCTCTTGCTGGAAAATAGAACAGCATTTGCAGAAGCGTTTGGCAATGTGC -4
GTCACAAGCCCACAGATATTTCCTGCTCCCCAGTGGATCGGGTGTAAACT--GAGCT----------CGGGAGCCTCTTGCTGGAAAATAGAACAGCATTTGCAGAAGCGTTTGGCAATGTGC *-8*
GTCACAAGCCCACAGATATTTCCTGCTCCCCAGTGGATCGGGTGTAAACT--GAGCT------------GGAGCCTCTTGCTGGAAAATAGAACAGCATTTGCAGAAGCGTTTGGCAATGTGC -10
GTCACAAGCCCACAGATATTTCCTGCTCCCCAGTGGATCGGGTGTAAACT--GAGCTTG---------------------CTGGAAAATAGAACAGCATTTGCAGAAGCGTTTGGCAATGTGC *-19*
GTCACAAGCCCACAGATATTTCCTGCTCCCCAGTGGATCGGGTGTAAACT--GAG-------------------CTCTTGCTGGAAAATAGAACAGCATTTGCAGAAGCGCTTGGCAATGTGC -17
GTCACAAGCCCACAGATATTTCCTGCTCCCCAGTGGATCGG-----------------------------TAGCCTCTTGCTGGAAAATAGAACAGCATTTGCAGAAGCGTTTGGCAATGTGC -27 ×2
GTCACAAGCCCACAGATATTTCCTGCTCCCCAGTGGATCGGGTGTAAACT--GAGCT---
--- -199
----------ACTGCAATTATTCAGGCCAAAGAATTCCTGGAAGGTGTTCAGGAGAAGGACAATGTTGTAGGGAGCCCAGAAGAGAAAATAAACAATCATGATGGTGAAGATAAGCCTCACAG
CR3 genome targeting insertion (8)
GTCACAAGCCCACAGATATTTCCTGCTCCCCAGTGGATCGGGTGTAAACT--GAGCTTGCTC-CGCTCGGGAGCCTCTTGCTGGAAAATAGAACAGCATTTGCAGAAGCGTTTGGCAATGTGC 1 ×4
GTCACAAGCCCACAGATATTTCCTGCTCCCCAGTGGATCGGGTGTAAACT--GAGCTTGCTCT-GCTCGGGAGCCTCTTGCTGGAAAATAGAACAGCATTTGCAGAAGCGTTTGGCAATGTGC 1
GTCACAAGCCCACAGATATTTCCTGCTCCCCAGTGGATCGGGTGTAAACT--GAGCTTGCTCA-GCTCGGTAGCCTCTTGCTGGAAAATAGAACAGCATTTGCAGAAGCGTTTGGCAATGTGC 1
GTCACAAGCCCACAGATATTTCCTGCTCCCCAGTGGATCGGGTGTAAACT--GAGCTTGCTCTCGCTCGGGAGCCTCTTGCTGGAAAATAGAACAGCATTTGCAGAAGCGTTTGGCAATGTGC 2 ×2
CR3 genome targeting deletion and insertion (1)
GTCACAAGCCCACAGATATTTCCTGCTCCCCAGTGGATCGGGTGTAAAC-AAGAG---------GCTCGGGAGCCTCTTGCTGGAAAATAGAACAGCATTTGCAGAAGCGTTTGGCAATGTGC (-1)/2/(-7)
