# Supplementary material for: CCR5 Gene Disruption via Lentiviral Vectors Expressing Cas9 and Single Guided RNA Renders Cells Resistant to HIV-1 Infection
Source: PLoS One. 2014 Dec 26;9(12):e115987. doi: 10.1371/journal.pone.0115987 (PMC4277423; doi:10.1371/journal.pone.0115987)
Supplement: S1 Table — Primer pairs used to amplify CCR5 gene target sites for T7EI assay and Sanger sequencing. (PDF) [file pone.0115987.s003.pdf]

	sgRNA	Target Sequence	Primer Code	Primer Sequence (5'-3')
T7EI	CR1	GCTTGTGACACGGACTCAAGTGG	CR1F990	TGCTGCATCAACCCCATCAT
			CR1R1750	CACAAGTCTCTCGCCTGGTT
	CR2	GGTCCTGCCGCTGCTTGTCATGG	CR2/3F593	GTTTGCGTCTCTCCCAGGAA
	CR3	GTAAACTGAGCTTGCTCGCTCGG	CR2/3R1254	CAGCCCAGGCTGTGTATGAA
	Intron-span		CR1/2/3F2559	GACAGGGAAGCTAGCAGCAA
			CR1/2/3R3893	CAGCCCAGGCTGTGTATGAA
Sanger Squencing	CR1	GCTTGTGACACGGACTCAAGTGG	CR1F1099	TTTTCCAGCAAGAGGCTCCC
			CR1R1254	CAGCCCAGGCTGTGTATGAA
	CR2	GGTCCTGCCGCTGCTTGTCATGG	CR2F651	ACCTGCAGCTCTCATTTTCCA
			CR2R820	GCCCTGTGCCTCTTCTTCTC
	CR3	GTAAACTGAGCTTGCTCGCTCGG	CR3F1061	AAAGCACATTGCCAAACGCT
			CR3R1203	TGCACAACTCTGACTGGGTC
	Intron-span		CR1/2/3F2559	GACAGGGAAGCTAGCAGCAA
			CR1/2/3R3893	CAGCCCAGGCTGTGTATGAA
