# Supplementary material for: CCR5 Gene Disruption via Lentiviral Vectors Expressing Cas9 and Single Guided RNA Renders Cells Resistant to HIV-1 Infection
Source: PLoS One. 2014 Dec 26;9(12):e115987. doi: 10.1371/journal.pone.0115987 (PMC4277423; doi:10.1371/journal.pone.0115987)
Supplement: S2 Table — The list of potential off- target sites that are highly homologous to CR2 or CR3 and prime pairs to amplify these potential off-target sites for T7EI assay. (PDF) [file pone.0115987.s004.pdf]

	Gene Name	Refseq NO.	Off Target Sequence	Primer Pairs (5'-3')
CR2	AKAP9	NM_147185	CCATGACAAGCAGGGG	ACAAGAGGAGTATGCTTGCCT<br>TTTCACTTGGTTGAGTTGCTCT
	ULK1	NM_003565	CCTGGACAAGCAGCGG	TGTCGTCCACTGTGAAGCAG<br>GGCACCCGTACAATCAAGGA
	MED16	NM_005481	CCGCTGCTTGTCCAGG	GAGACCTGCCTGGAAGCAAA<br>CCCCACTGCTCACTTTGGAT
CR3	CLPP	NM_006012	CCTAGCGAGCGAGCTC	TCGGTAAGGGGACTCGAACT<br>GATGGGAATGAGCGGGAGAG
	ASB9P1	NR_033769	GAGCTTGCTCGCTTAG	GCCTCCAGCCAGTTAGACC<br>GGTTCAGGTCTGCTCCTGAC
	LINC00265	NR_026999	GAGCTTGCTTGCTGGG	GATGCCCAACTCATGCCTCT<br>ATTTCCAGGCCGACTTGAGG
	SH2D5	NM_001103161	GAGCTTGCTGGCTGGG	CCACTCACATGAGGTCCCAC<br>GGCCACTGACTTGCTTTGTG
	NR2F1	NM_005654	GAGCTTGCTCGCCGGG	CCGGCGTGAATTATCCCGTA<br>TGTTGTGGTTCCTGCTGTTT
	PRRT1	NM_030651-2	GAGCTTGCTCGCAAGG	ATCCCTAAAAACGCCCCTGG<br>CCAGGGAGAAGGGTCCTACA
	ASB9P1	NM_024087	CTAAGCGAGCAAGCTC	GCCTCCAGCCAGTTAGACC<br>GGTTCAGGTCTGCTCCTGAC
	ENDOV	NM_001164638	CCTAGGGAGCAAGCTC	ACTCCAAAGCAAAGCCCAGT<br>TTCTACGCTTGCACTGCTCC
	SOBP	NM_018013	CCTGGCGAGCAAGCTC	TGGCACTGAGTTGGTGAGAC<br>CACCAGTCAGGGAGAGGAGA
